# Cancer-type dependent expression of CK2 transcripts

**DOI:** 10.1371/journal.pone.0188854

**Published:** 2017-12-04

**Authors:** Melissa M. J. Chua, Migi Lee, Isabel Dominguez

**Affiliations:** Department of Medicine, Boston University School of Medicine, Boston MA, United States of America; Columbia University, UNITED STATES

## Abstract

A multitude of proteins are aberrantly expressed in cancer cells, including the oncogenic serine-threonine kinase CK2. In a previous report, we found increases in *CK2* transcript expression that could explain the increased CK2 protein levels found in tumors from lung and bronchus, prostate, breast, colon and rectum, ovarian and pancreatic cancers. We also found that, contrary to the current notions about CK2, some *CK2* transcripts were downregulated in several cancers. Here, we investigate all other cancers using Oncomine to determine whether they also display significant CK2 transcript dysregulation. As anticipated from our previous analysis, we found cancers with all *CK2* transcripts upregulated (e.g. cervical), and cancers where there was a combination of upregulation and/or downregulation of the *CK2* transcripts (e.g. sarcoma). Unexpectedly, we found some cancers with significant downregulation of all *CK2* transcripts (e.g. testicular cancer). We also found that, in some cases, *CK2* transcript levels were already dysregulated in benign lesions (e.g. Barrett’s esophagus). We also found that *CK2* transcript upregulation correlated with lower patient survival in most cases where data was significant. However, there were two cancer types, glioblastoma and renal cell carcinoma, where *CK2* transcript upregulation correlated with higher survival. Overall, these data show that the expression levels of *CK2* genes is highly variable in cancers and can lead to different patient outcomes.

## Introduction

In humans, there are two *CK2* kinase genes, *CSNK2A1* and *CSNK2A2*, that code for highly-conserved serine/threonine kinase proteins, CK2α and CK2α*’*, respectively. CK2α and CK2α’ differ in their C-terminal sequence [1–4]. They also differ in their expression pattern and the phenotype of knockout in mice. Thus, CK2α has higher levels and more widespread expression in mouse tissues than CK2α’ (mostly brain and testis) [5]. *CK2α* deficient mice die at mid-gestation while *CK2α’* deficient mice are viable albeit males are sterile, suggesting that they have different functions that cannot be compensated by the other protein [5, 6]. CK2 kinases can function as monomeric kinases, and also within a tetrameric complex composed of two CK2 kinase proteins (CK2α and/or CK2α’) and two regulatory proteins with no enzymatic activity (CK2β, coded by *CSNK2B*). Within this tetrameric complex, CK2β alters CK2 kinase substrate specificity [7]. Additionally, an intronless *CK2α* pseudogene (CK2aP, coded by *CSNK2A1P/CSNK2A3*) [8] codes for a predicted non-coding RNA that is relevant in human cancer [9].

CK2 has been implicated in cancer in humans and mice [10–14]. CK2 proteins are upregulated in the human tumors tested so far, suggesting a role in cancer progression (Reviewed in [15, 16]). Indeed, overexpression of CK2α in mammary gland and the lymphoid compartment leads to tumors in mice [17–20]. In cancer, CK2 is proposed to regulate essential cellular processes such as cell growth [21], cell proliferation [22, 23],cell survival [24, 25], cell morphology [26, 27], cell transformation [12, 13] and angiogenesis [28]. Importantly, CK2 protein upregulation and/or nuclear localization correlate with cancer clinicopathology and prognosis in some cancers (e.g. gastric cancer [29, 30], and head and neck cancer [31–33]). These data suggest that CK2 is a target for cancer therapy and hence, several CK2 inhibitors are being tested in clinical trials (reviewed in [16, 34, 35]).

The importance of *CK2* transcripts in cancer is also being investigated. The original view in the literature is that CK2 is predominantly regulated post-transcriptionally, however, recent studies strongly suggest that regulation at the transcriptional level is also important in some cancers ([9], and references within). Unpredictably, some cancers show underexpression of *CK2* transcripts (e.g. *CK2α’* in breast, ovarian, and pancreatic cancer)[9]. Importantly, recent studies show that *CK2* transcripts could have a diagnostic value (e.g. *CK2α* and *CK2α’* in renal cell carcinoma [36]*; CK2β* in invasive ductal and lobular breast carcinoma [9]). Furthermore, *CK2* transcript levels could have a prognostic value in cancers (e.g. *CK2α* in squamous cell carcinoma of the lung [9, 37]). For the most part, high levels of *CK2* transcript correlate with lower overall survival (e.g. breast and ovarian cancer [9], glioblastoma, kidney and liver cancer [38–40]). However, in lung adenocarcinoma, high levels of *CK2α’* and *CK2αP* correlate with higher survival rates [9]. Overall, these data indicate the need to determine the extent to which *CK2* genes could be significantly up- or down-regulated in other cancers not studied so far, and raise the question of whether in these other cancers *CK2* gene expression levels correlated with overall patient survival. Therefore, using Oncomine, we analyzed the expression levels of *CK2* transcripts in bladder, central nervous system (CNS), cervical, esophageal, gastric, head and neck, kidney, blood (leukemia, non-Hodgkin lymphoma, monoclonal gammopathies), liver, mesothelioma, parathyroid, sarcoma, skin, and testicular cancers. We also analyzed the correlation between *CK2* gene expression and overall patient survival to determine whether it has prognostic value, in cancers where data was available.

## Methods

### Information on cancer types, prevalence and treatment

To retrieve this information, we used the NCI (http://www.cancer.gov), ACS (http://www.cancer.org) and CDC (http://www.cdc.gov) web sites.

### Oncomine analysis

The transcript level of *CK2* genes and pseudogene (*CSNK2A1*, *CSNK2A2*, *CSNK2B*, and *CSNK2A1P/CSNK2A3*) was analyzed using the Oncomine database (www.oncomine.org, July 2017, Compendia Bioscience, Ann Arbor, MI) [41]. This database contains different datasets, each containing the data from a single publication. We used datasets that compared cancer *vs*. normal patient specimens for the different cancer types. We used the default view, where all the expression data included is obtained before cancer treatment. Oncomine uses *t*-test statistics to compare the means of gene expression to determine whether a gene is significantly over or underexpressed in tumors compared to normal tissue. Each Oncomine search provides the p-value (probability that there is a true difference in gene expression), fold change (the difference between the mean values of the classes that are being compared), and rank (where genes are ranked by their P-value; the one’s at the top % have more significant P-values that other genes) for each gene. The data we obtained were represented in tables where #Samples represents the total number of samples in the dataset (controls and tumor samples), and Reference is the original publication of the data. To reduce false discovery rate, p < 0.01, fold change > +/- 1.5 were selected as thresholds. In the tables, we have included all the data that matched these thresholds. We have also included a few data that were significant but were below the 1.5-fold change threshold (up to approx. 1.3), as they indicate low variance. If a particular *CK2* gene was not tested in the original publication, we have written it in the text and also noted in the final summary table as “-“. *CK2* genes that were tested but found non-significant were not mentioned in the text, but noted as “n.s.” in the final summary table.

### Kaplan-Meier analysis

The prognostic value of the expression of *CK2* transcripts in gastric cancer was analyzed using the Kaplan-Meier Plotter (http://kmplot.com/analysis), a database that integrates gene expression and clinical outcomes [42]. At present, Kaplan-Meier Plotter contains information on survival outcomes for 1,065 gastric cancer patients in relation to the expression levels of the 22,277 genes in their database [43]. The best specific probes (JetSet probes) were selected for each *CK2* transcript. Patients were split into two groups, high versus low expression levels of each *CK2* gene (based on the median expression), and the overall survival rates of these two patient groups were compared. To reduce false discovery rate, we selected p < 0.01 as a threshold. Hazard ratio with 95% confidence intervals and log rank p value were also calculated.

The prognostic value of expression of the *CK2* transcripts in other cancers was analyzed using data from the web sites of the University of California Santa Cruz (UCSC) Xena (https://genome-cancer.soe.ucsc.edu/proj/site/xena/heatmap/) and Cell Index (CellX) (http://54.149.52.246/cgi-bin/RPPA/cellx.cgi). These data repositories contain overall survival (OS) and recurrence free survival (RFS) information based upon data generated by the TCGA Research Network (http://cancergenome.nih.gov/). The data from these two databases were combined to make one complete data set with as many patient data as possible. We found information for the following cancers: cervical cancer, acute myeloid leukemia, bladder cancer, kidney papillary cell carcinoma, esophageal cancer, glioblastoma, head and neck cancer, kidney chromophobe, kidney clear cell carcinoma, liver cancer, large B-cell lymphoma, melanoma, mesothelioma, sarcoma, stomach cancer, and testicular cancer.

Kaplan-Meier survival curves and statistical analysis was performed for the *CK2* transcripts for each cancer type by the Department of Medicine Biostatistics consultants, using SAS 9.3 (SAS institute, Cary, NC, USA). For each transcript and tumor type, the cancer patients were stratified into two groups: high and low transcript expression levels based on whether the expression of the gene was above or below the median expression of the gene. The Log-Rank test was used to compare the overall survival curves of the over-expression and under-expression patient groups, and a p-value obtained. For our analysis, alpha equal to or below 0.05 was chosen as significant. We used R Studio (R Studio, Boston, MA, USA) to represent the Kaplan Meier survival curves for the cancers with significant p-values in the Log-Rank test. We also included in the figures the hazard ratio (HR) and the confidence interval (CI) from the Cox proportional hazards model. The cancers for which none of the *CK2* transcripts was significantly associated with patient overall survival were Leukemia (151 patients), Bladder (407 patients), Kidney (320 patients), Esophageal (184 patients), Kidney Chromophobe (65 patients), Lymphoma (47 patients), Melanoma (251 patients), Stomach (387 patients) and Testicular (134 patients).

## Results and discussion: CK2 transcript expression levels and correlation with overall patient survival by cancer type

Here, the different cancer types studied are organized alphabetically expect for liver cancer, which is placed after all the blood cancers. To simplify the reading, we have chosen to use the terms *CK2α*, *CK2α*’, *CK2β* and *CK2αP* to refer to the genes *CSNK2A1*, *CSNK2A2*, *CSNK2B*, *CSNK2A1P/CSNK2A3*, respectively.

### Bladder cancer

Transitional cell carcinomas (also known as urothelial carcinomas) make up about 90% of all bladder cancers, and originate in cells of the inner lining of the bladder. Transitional cell carcinomas are clinically subdivided into superficial (non-muscle invasive) and invasive tumors. Less common bladder cancer types include squamous cell carcinoma and adenocarcinoma. There is no standard or routine screening test for bladder cancer, leading to a low rate of early diagnosis. Treatments for bladder cancer include surgery, radiation therapy, chemotherapy, and biological therapy.

#### CK2 in bladder cancer

Oncomine analysis revealed significant overexpression of all three *CK2* transcripts in both superficial and invasive types of bladder transitional cell carcinoma (Table 1). In line with our findings, Zhang *et al*. found overexpression of CK2α transcript in transitional cell carcinomas (subtype not specified) compared with adjacent normal tissue and also in transitional cell carcinoma cells lines compared with normal urinary epithelial cell lines [44]. There were no data in Oncomine for squamous cell carcinoma and adenocarcinoma, possibly due to their low frequency in the population.

**Table 1:** Analysis of changes in *CK2* gene expression in bladder cancer. P-values, fold change, rank and datasets are shown.

Gene	p-value	Fold Change	Rank (Top %)	Dataset	#Samples	Reference
**Superficial Bladder Carcinoma**						
*CK2α*	2.26 10^−14^	2.044	5%	Sanchez-Carbayo Bladder 2	157	[47]
	3.70 10^−10^	1.635	1%	Dyrskjot Bladder 3	60	[48]
*CK2α’*	4.17 10^−6^	1.775	9%	Dyrskjot Bladder 3	60	[48]
	1.24 10^−5^	1.635	24%	Sanchez-Carbayo Bladder 2	157	[47]
*CK2β*	2.65 10^−17^	2.874	3%	Sanchez-Carbayo Bladder 2	157	[47]
	7.38 10^−8^	2.010	3%	Dyrskjot Bladder 3	60	[48]
**Invasive Bladder Carcinoma**						
*CK2α*	9.15 10^−8^	1.696	7%	Sanchez-Carbayo Bladder 2	157	[47]
	2.33 10^−5^	1.583	7%	Dyrskjot Bladder 3	60	[48]
*CK2α’*	0.003	1.501	25%	Dyrskjot Bladder 3	60	[48]
*CK2β*	1.48 10^−7^	2.211	1%	Dyrskjot Bladder 3	60	[48]
	0.001	1.420	21%	Sanchez-Carbayo Bladder 2	157	[47]

Elevated levels of CK2α protein are also found in bladder cancer. Thus, Zhang *et al*. found increased levels of CK2α protein in bladder carcinomas (subtype not specified) compared with adjacent normal tissue, and in transitional cell carcinoma cells lines compared with normal urinary epithelial cell lines [44]. High staining of CK2α protein is detected in invasive bladder transitional cell carcinoma but not in low grade non-invasive tumors [45]. In addition, CK2α was increased in the lumen of exosomes of metastatic cells versus non-metastatic bladder cancer cells [46]. CK2α protein staining positively correlates with histological grade but not with tumor size, tumor stage or gender [44]. Kaplan–Meier analysis does not reveal an association between high CK2α staining and survival. We further reviewed the role of CK2 proteins in bladder cancer in [16]. All together, these data suggest that CK2α protein, but perhaps not the transcript, could be used as a diagnostic marker in bladder cancer.

### Central nervous system (CNS) cancer

Overall (among children and adults), the most common CNS cancer type is glioma (33%), which is subclassified into astrocytomas (20% of total CNS cancer), oligodendrogliomas (2% of total CNS cancer), ependymomas (2% of total CNS cancer), and mixed gliomas. A high-grade (IV) astrocytoma is known as glioblastoma. However, among adults only, the most common CNS cancers is meningioma (33%). Other CNS tumor types include, but are not limited to, medulloblastomas, gangliogliomas, schwannomas, and craniopharyngiomas. While the rate of diagnosis at an early stage is relatively high, with 76.6% of patients diagnosed at the local stage, the 5-year survival for localized CNS cancer is only 36.3%. There are currently no screening tests for CNS cancers. Standard treatments involve watchful waiting, surgery, radiation therapy, chemotherapy, and targeted therapy.

#### CK2 in CNS cancer

Oncomine analysis revealed overexpression and underexpression of *CK2α* transcripts in astrocytoma and glioblastoma (Table 2). In agreement with our data, other publications also show under- and over-expression of CK2α transcripts. For example, Zheng *et al*. show *CK2α* transcripts mostly overexpressed but also underexpressed in glioblastomas from the TCGA [49]. They attribute increases in *CK2α* transcripts to gene dosage gains of *CSNK2A1*. Nitta et al. and Ladha *et al*. also find increased (mostly) and decreased *CK2α* transcripts in glioblastomas [38, 50]. Dubois *et al*. find heterogeneous increases in *CK2α* transcripts in glial brain tumors (oligodendrogliomas, astrocytomas and glioblastoma), but, in this case, *CK2* transcript levels did not correlate with gene amplification, suggesting transcriptional mechanisms at play [51]. This heterogeneity in the levels of *CK2α* transcripts in these cancers may be resolved by increasing the sample size, to determine whether over- or under- expression correlate with any clinicopathological or demographic variables.

**Table 2:** Analysis of changes in *CK2* gene expression in CNS cancer. P-values, fold change, rank and datasets are shown.

Gene	p-value	Fold Change	Rank (Top %)	Dataset	#Samples	Reference
**Astrocytoma**						
*CK2α*	0.001	1.352	8%	Shai Brain	42	[53]
	0.003	-1.322	9%	Sun Brain	180	[58]
*CK2β*	3.12 10^−4^	1.518	5%	Shai Brain	42	[53]
**Glioblastoma (Astrocytoma Grade IV)**						
*CK2α*	2.02 10^−6^	1.370	15%	Sun Brain	180	[58]
	2.33 10^−4^	1.294	11%	Shai Brain	42	[53]
	9.21 10^−4^	4.072	13%	Bredel Brain 2	54	[59]
	0.01	-2.382	42%	Lee Brain	101	[52]
*CK2α’*	2.59 10^−4^	-2.132	23%	Lee Brain	101	[52]
	8.13 10^−4^	-1.314	17%	Bredel Brain 2	54	[59]
*CK2β*	1.52 10^−7^	1.440	2%	Shai Brain	42	[53]
	1.19 10^−5^	-2.338	14%	Lee Brain	101	[52]
**Anaplastic Oligodendroglioma (grade III)**						
*CK2α’*	1.15 10^−6^	1.637	3%	French Brain	33	[60]
*CK2β*	0.001	1.499	15%	French Brain	33	[60]
**Anaplastic Oligoastrocytoma (oligodendroglioma + astrocytoma)**						
*CK2α*	0.008	1.686	12%	French Brain	33	[60]
*CK2α’*	0.004	-1.624	14%	Bredel Brain 2	54	[59]
*CK2β*	0.003	1.569	8%	French Brain	33	[60]

Oncomine analysis showed overexpression of *CK2α’ in* oligodendrogliomas and astrocytomas, and over-and under-expression of *CK2α’* transcripts in glioblastoma. In agreement with our data, Dubois *et al*. find elevated levels of *CK2α’* transcripts in oligodendrogliomas, astrocytomas, and the majority of glioblastomas. They also found *CK2α’* gene dosage loss in glioblastoma (1/18 samples) [51].

We found overexpression of *CK2β* transcripts in CNS cancers as detailed in Table 2, except in glioblastoma where there was conflicting data [52, 53]. We could not find information on demographic or clinicopathological characteristic that could further explain this discrepancy. Published data also show heterogeneous expression of *CK2β* transcripts. Thus, some studies find *CK2β* transcripts mostly unchanged [38] while others find *CK2β* transcripts mostly upregulated in glial brain tumors (oligodendrogliomas, astrocytomas and glioblastoma) [51]. Further studies will help determine to determine whether over- or under- expression correlate with any clinicopathological or demographic variables. Interestingly, the *CK2β* gene is deleted in a small percent of glioblastomas (7%, in Zheng *et al*., and 1/18 in Dubois *et al*.)[49] Dubois, 2016 #532], but gene dosage gains were also found in a small percent of glioblastomas (1/18 samples) [51]. If after further confirmation, if *CK2α’* and/or *CK2β* are found to be downregulated in some subtypes of glioblastoma (grade IV astrocytoma), they could have diagnostic value for these subtypes and also to distinguishing high from low grade astrocytomas that have elevated levels of both transcripts. For other CNS cancer types, Oncomine analysis showed no significant findings for any of the *CK2* transcripts. There was no data for *CK2αP* in astrocytoma.

Regarding CK2 proteins in CNS cancer, Dixit *et al*. (n = 5) find increased levels of CK2α protein in glioblastomas (5/5 samples), while Nitta *et al*. find increased (4/7 samples) and decreased (1/7 samples) levels CK2α protein [38, 54]. There was a correlation between increased levels of CK2α protein and transcript levels in glioblastomas [38]. In addition, CK2 kinase activity as also higher in most glial brain tumors (oligodendrogliomas, astrocytomas and glioblastoma) but did not correlate neither with transcript levels nor gene amplification [51]. These data suggest that translational regulatory mechanisms, post-translational modifications or different levels of CK2β protein (not measured in these studies) could play a role in the increased CK2 activity observed.

The increase in CK2 activity found in glial brain tumors does not correlate with tumor grade (II, III or IV), and, in the case of glioblastomas, with tumor subtype [51]. However, when CK2 subcellular localization is analyzed, there is a correlation between increased cytoplasmic staining of CK2α with increasing grades of malignancy (grades II, II and IV (glioblastoma)), suggesting that cytoplasmic CK2α protein level has diagnostic value [50]. In contrast, the same study find astrocytomas of all grades show a decrease in nuclear CK2α protein staining compared to control samples. However, other publications show increased levels of both nuclear and cytoplasmic CK2α in CNS cancer. For example, glioblastoma samples show higher staining of CK2α either in the cytoplasm or in both cytoplasm and nucleus, compared with normal brain tissue [51]; and CK2α protein is elevated in cytoplasm and nucleus in grade II, III and IV gliomas (no subtype information) [55]. As for *CK2* transcripts, further studies are needed to determine whether over- or under- expression of CK2α proteins correlate with any clinicopathological or demographic variables.

**Fig 1:**
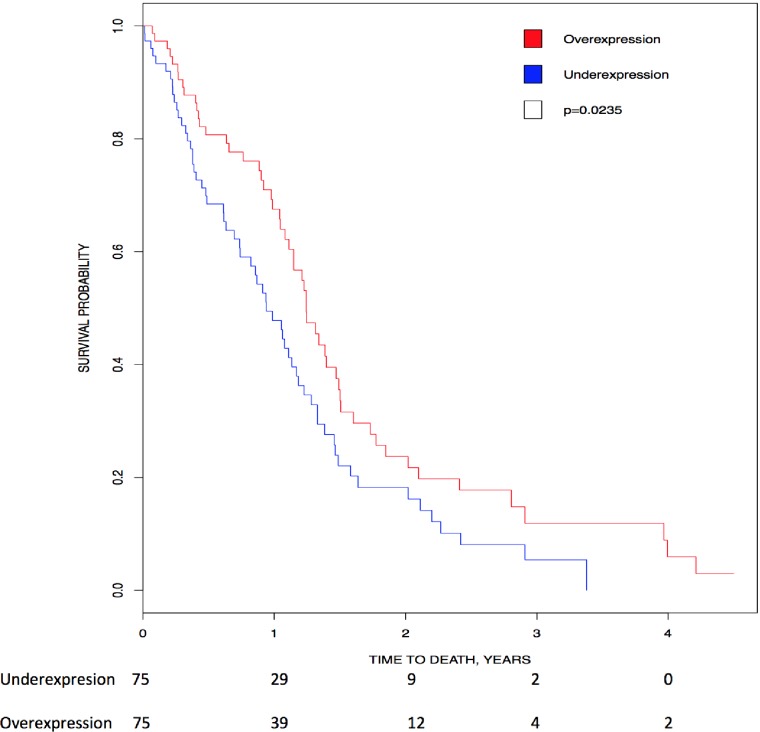
Correlation of *CK2* gene expression to overall patient survival in glioblastoma. Patients were stratified into above-median (red line) and below-median (blue line) expression for *CK2α*. Kaplan Meier analysis show that high levels of *CK2α* transcript correlated with higher survival (HR = 0.65, 95% CI [0.446, 0.947], p = 0.0235).

Unexpectedly, Kaplan-Meier analysis showed high expression of *CK2α* transcripts directly associated with higher overall survival in glioblastoma (p = 0.0235)(Fig 1). In contrast with our analysis, Nitta *et al*. show high expression of *CK2α* transcripts correlating with lower survival in mesenchymal glioblastoma, but not in the other types of glioblastomas (classical, neural and proneural) using the TCGA database [38]. In addition, using the Repository of Molecular Brain Neoplasia Data (Rembrandt) they found a trend where high *CK2α* transcript correlated with poor prognosis [38]. We do not have enough information to explain the difference between our analysis and that of Nitta *et al*. [38]. As for CK2α protein, there is no difference in patient survival between high staining of CK2α in the cytoplasm or in both cytoplasm and nucleus [51], therefore we may not be able to use CK2α protein upregulation as a prognostic factor in glioblastoma.

Mounting pre-clinical evidence suggests that CK2 inhibitors could be effective in glioblastoma [16]. An additional pre-clinical model show significantly improved survival of mice treated with a combination of TMZ (temozolomide, the standard chemotherapy for glioblastoma) and CK2 inhibitor CX-4945 when compared to TMZ alone (*p* <0.05) This effect occurs only when drugs are administered simultaneously every 6 days [56]. In addition, inhibition of CK2 with CX-4945 and TBB leads to decreased glioblastoma invasion in glioblastoma cell lines [57].

### Cervical cancer

Cervical cancer is the 2^nd^ leading cause of death among women worldwide, and most commonly caused by human papillomavirus (HPV) infection. Most cases of cervical cancer are preventable with the HPV vaccine. Despite well-established screening tests in place, only 46.9% of patients are diagnosed at the localized stage. Standard treatments involve surgery, radiation therapy, and chemotherapy.

#### CK2 in cervical cancer

Oncomine analysis revealed significant overexpression of all three *CK2* genes in cervical cancer (Table 3). Oncomine had no data on *CK2αP* expression in cervical cancer.

**Table 3:** Analysis of changes in *CK2* gene expression in cervical cancer. P-values, fold change, rank and datasets are shown.

Gene	p-value	Fold Change	Rank (Top %)	Dataset	#Samples	Reference
**Cervical Cancer**						
*CK2α*	7.37 10^−5^	1.539	19%	Pyeon Multi-cancer	84	[63]
*CK2α’*	8.59 10^−7^	1.914	9%	Pyeon Multi-cancer	84	[63]
*CK2β*	3.98 10^−4^	1.766	25%	Pyeon Multi-cancer	84	[63]

**Fig 2:**
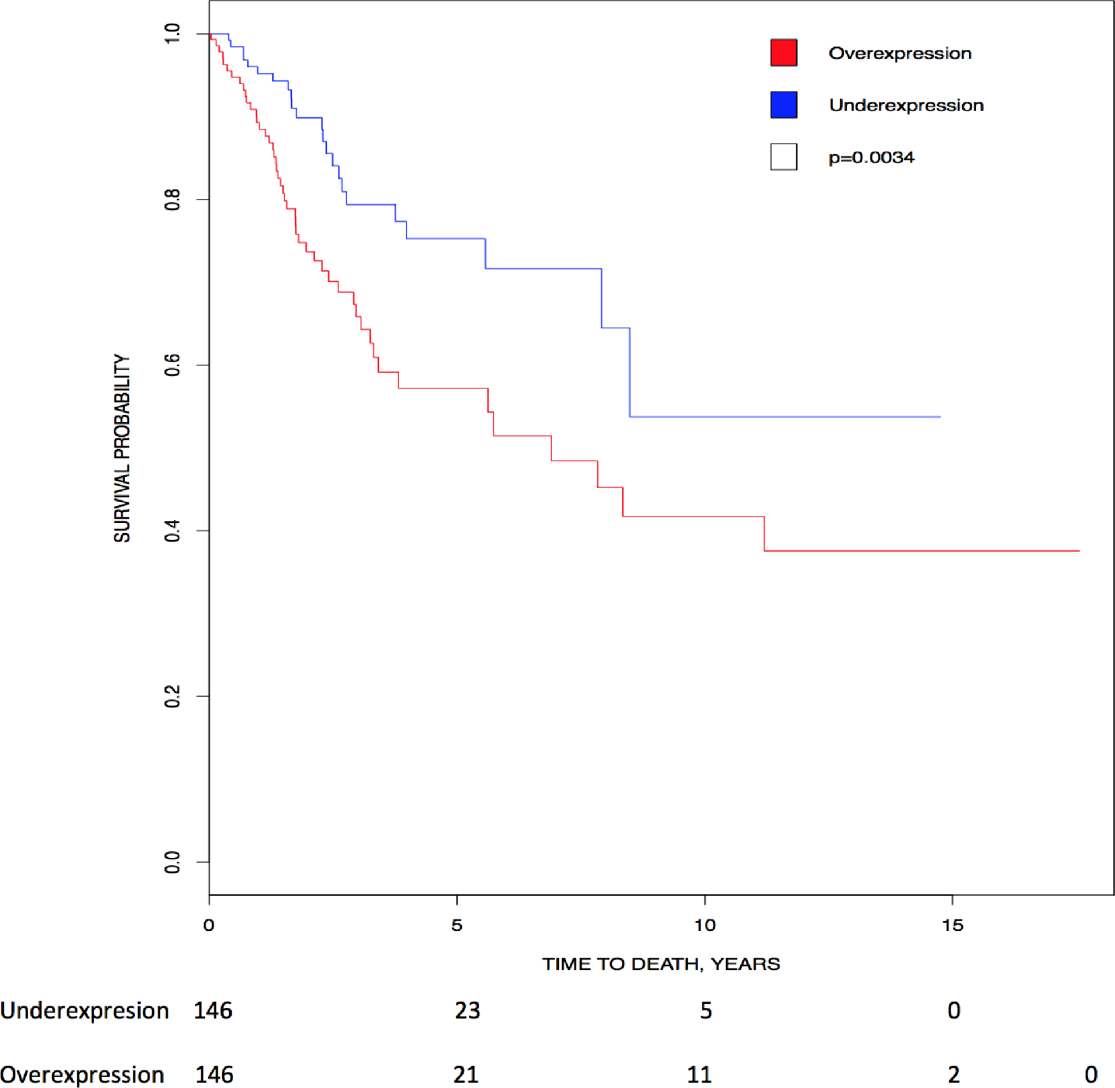
Correlation of *CK2* gene expression to overall patient survival in cervical cancer. Patients were stratified into above-median (red line) and below-median (blue line) expression of *CK2αP*. Kaplan Meier analysis show that high levels of *CK2αP* transcript correlated with lower survival (HR = 2.075, 95% CI [1.260, 3.418]; p = 0.0034).

Kaplan-Meier analysis showed that higher expression of *CK2αP* transcripts directly correlated with lower survival, suggesting its prognostic value in cervical cancer (p = 0.0034)(Fig 2).

Interestingly, CK2 activity is elevated in HPV-immortalized human keratinocytes and cervical and oral carcinoma cell lines compared to parental cell lines and normal cervical and tongue keratinocytes [61]. In HPV-immortalized cell lines, CK2 activity is highest during the G1 phase of the cell cycle [61]. During G1, CK2 seems to phosphorylate and activate the HPV type 18 E7 protein, a transcription factor that induces cellular proliferation by promoting S-phase entry [61, 62]. Based on these data, it is plausible that CK2 stimulates HPV-linked cervical cancer by promoting S-phase entry of the cell cycle. Importantly, CK2 inhibitors are being tested in cervical cancer in clinical trials (reviewed in [16]).

### Esophageal cancer

The two most common types of esophageal cancer are adenocarcinoma (most common in USA) and squamous cell carcinoma (most common worldwide). Adenocarcinoma forms in glandular cells close to the stomach, and squamous cell carcinoma forms in flat cells of the upper and middle esophagus. Esophageal cancer is associated with aging, and alcohol and tobacco use (only squamous cell carcinoma), and affects more men than women across all racial and ethnic groups. Importantly, the risk for esophageal adenocarcinoma is increased in Barrett’s esophagus, a condition characterized by replacement of the esophageal tissue by tissue similar to that of intestinal lining that occurs in individuals with long-term gastroesophageal reflux disease. Procedures to detect or diagnose esophageal cancer include physical examination, upper endoscopy, chest x-ray, and a barium swallow test, but only 20.5% of patients are diagnosed at the local stage. Treatment options include surgery, radiation therapy, chemotherapy, chemoradiation therapy, laser therapy, and electrocoagulation.

#### CK2 in esophageal cancer

Oncomine analysis showed significant downregulation of all *CK2* genes in Barrett’s esophagus and esophageal adenocarcinoma (Table 4). In esophageal adenocarcinoma, there was conflicting data for *CK2α*’ transcripts, where the Kim *et al*. study showed a downregulation while the Hao *et al*. study showed an upregulation [64, 65]. In both studies, age ranges are similar, and the patients were from the USA. The Kim *et al*. study included 90.7% male, average age 61.82, and 49.2% white patients; the Hao *et al* study included 94.1% male, average age 66.5±11.7, and no ethnicity was described. We do not have enough data to determine if clinicopathological characteristics (only Kim *et al*. study includes stages I to IV cancer) explain the discrepancy in the results.

**Table 4:** Analysis of changes in *CK2* gene expression in esophageal cancer. P-values, fold change, rank and datasets are shown.

Gene	p-value	Fold Change	Rank (Top %)	Dataset	#Samples	Reference
**Barrett’s Esophagus**						
*CK2α*	5.27 10^−10^	-2.356	6%	Kim Esophagus	118	[64]
*CK2α’*	1.36 10^−4^	-1.660	23%	Kim Esophagus	118	[64]
*CK2β*	2.53 10^−6^	-1.935	15%	Kim Esophagus	118	[64]
	2.65 10^−7^	-1.333	2%	Wang Esophagus	52	[67]
*CK2αP*	5.63 10^−10^	-1.578	6%	Kim Esophagus	118	[64]
**Esophageal Adenocarcinoma**						
*CK2α*	2.86 10^−9^	-1.805	12%	Kim Esophagus	118	[64]
	0.012	-1.334	15%	Kimchi Esophagus	24	[68]
*CK2α’*	1.37 10^−6^	-1.591	19%	Kim Esophagus	118	[64]
	3.35 10^−4^	2.524	7%	Hao Esophagus	48	[65]
*CK2β*	4.9 10^−9^	-2.042	13%	Kim Esophagus	118	[64]
*CK2αP*	1.82 10^−7^	-1.315	17%	Kim Esophagus	118	[64]

In addition, Chen *et al*. find both overexpression (6/8) and underexpression (2/8) of *CK2β* transcripts also in esophageal carcinoma, compared with adjacent normal mucosal tissue [66]. They also find elevated CK2β staining in 86% of tumors levels while the rest have none, weak or moderate staining, and CK2β protein levels are elevated in ¾ samples. Furthermore, they find that *CK2β* transcript expression level correlated with cancer stage (I, II and III) therefore, *CK2β* transcripts could have diagnostic value [66].

### Gastric cancer

The most common type of gastric cancer is adenocarcinoma, accounting for 95% of all gastric cancers, and develops from cells forming the mucosa (innermost lining) of the stomach. Other types can include carcinoid tumor and gastrointestinal stromal tumor. Gastric cancer is more common in men than women, and whites have the lowest rates of gastric cancer compared to all other racial/ethnic groups in the USA. A number of factors increase gastric cancer risk including *Helicobacter pylori* (*H*. *pylori*) infection of the stomach, smoking, family history of gastric cancer, high-salt diet or smoked foods, and low intake of fruits and vegetables. There is currently no standard or routine screening test for gastric cancer. Treatments include surgery, chemotherapy, radiation therapy, and chemoradiation.

#### CK2 in gastric cancer

Oncomine analysis revealed overexpression of *CK2α* transcripts in all types of gastric cancer found in Oncomine; some types also showed overexpression of *CK2αP* and *CK2β* transcripts (Table 5). *CK2α’* transcripts showed a downregulation in diffuse gastric adenocarcinoma. However, there were conflicting findings for *CK2α’* in gastric intestinal type adenocarcinoma, with the Cho *et al*. study showing decreased expression and the D’Errico *et al*. study showing overexpression [69, 70]. The studies have similar demographics except for country of origin: South Korea, 63.2% female, mean age 62 (32–83 years) *versus* Italy, 60% female, mean age 74 (61–89 years) respectively. We do not have enough data to determine if clinicopathological characteristics (only D’Errico *et al*. study included stage III cancer) explain the discrepancy in the results.

**Table 5:** Analysis of changes in CK2 gene expression in gastric cancer. P-values, fold change, rank and datasets are shown.

Gene	p-value	Fold Change	Rank (Top %)	Dataset	#Samples	Reference
**Gastric Intestinal Type Adenocarcinoma**						
*CK2α*	1.98 10^−10^	1.611	5%	Chen Gastric	132	[72]
	7.64 10^−8^	1.661	8%	DErrico Gastric	69	[69]
*CK2α’*	5.68 10^−5^	1.473	20%	DErrico Gastric	69	[69]
	4.75 10^−4^	-1.454	5%	Cho Gastric	90	[70]
*CK2β*	4.56 10^−7^	1.761	11%	DErrico Gastric	69	[69]
*CK2αP*	1.34 10^−5^	1.346	16%	Chen Gastric	132	[72]
**Diffuse Gastric Adenocarcinoma**						
*CK2α*	6.9 10^−5^	1.540	5%	Chen Gastric	132	[72]
*CK2α’*	2.95 10^−4^	-1.549	6%	Cho Gastric	90	[70]
**Gastric Mixed Adenocarcinoma**						
*CK2α*	0.011	2.136	19%	Chen Gastric	132	[72]
	0.012	1.449	17%	Cho Gastric	90	[70]

CK2*α* and CK2β protein levels are elevated in gastric carcinoma (2/2 samples) compared to non-tumor tissue [30]. As for subcellular localization, CK2*α* protein staining (nuclear and cytoplasmic) is elevated in gastric carcinoma and dysplastic lesions compared to normal mucosa [29], and CK2β staining is also elevated in gastric carcinoma (nuclear and cytoplasmic) [30].

**Fig 3:**
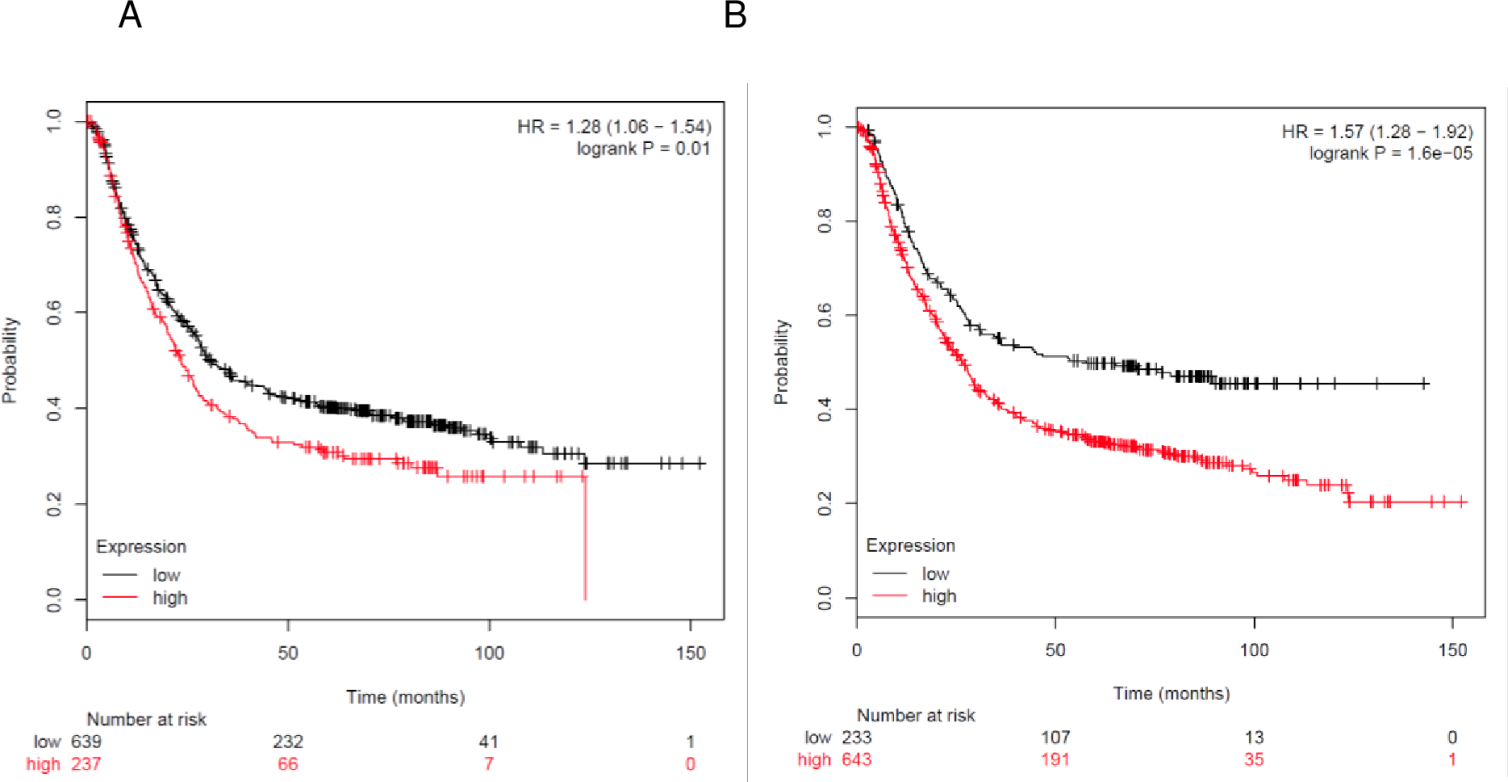
Correlation of *CK2* gene expression to overall patient survival in gastric cancer. Patients were stratified into above-median (red line) and below-median (black line) expression of *CK2*. (A) High levels of *CK2α* transcript correlated with lower survival (HR = 1.28, 95% CI [1.06, 1.54]; p = 0.01). (B) High levels of *CK2β* transcript correlated with lower survival (HR = 1.57, 95% CI [1.28, 1.92]; p = 1.6 10^−5^).

Kaplan-Meier Plotter analysis showed that higher expression of *CK2α* (p = 0.010) and *CK2β* (p = 1.6 10^−5^) transcripts directly correlated with lower survival in gastric cancer patients (Fig 3). In addition, published results show that overexpression of CK2α protein correlates with poor survival [29], and elevated levels of nuclear CK2β correlated with poor survival in gastric cancer [30]. Therefore, both CK2α and CK2β transcripts and proteins could be prognostic markers for gastric carcinoma.

CK2α protein was found also to be an independent diagnostic indicator for gastric carcinoma, as nuclear levels of CK2α were associated with higher tumor stage, presence of lymph node metastasis, presence of venous invasion and tumor invasion [29]. Additionally, elevated levels of nuclear CK2β correlated with depth of invasion [30]. These data show that increased CK2α and CK2β protein levels can be correlated with the invasive potential of gastric cancer cells. Indeed, CK2 inhibition led to a decreased migration and invasion of gastric epithelial cells and epithelial-to-mesenchymal transition phenotype, a hallmark for metastasis [29, 71]. At least two mechanisms are described to contribute to *CK2α*’s association with gastric carcinoma; through phosphorylation and activation of the protein “deleted in breast cancer 1” (DBC1) [29], and through facilitation of *H*. *pylori’*s effects on migration and invasion [71]. Overall, both CK2α and CK2β transcripts and proteins have prognostic value in gastric cancer, and CK2α and CK2β proteins have diagnostic value.

### Head and neck cancer

Head and neck cancer encompasses cancers in the oral cavity, pharynx, larynx, paranasal sinuses and nasal cavity and salivary glands; 90% arise from squamous cells (e.g. tonsillar carcinoma, tongue carcinoma, floor of the mouth carcinoma, and oropharyngeal carcinoma). The two highest risk factors for head and neck cancer are alcohol and tobacco use. Other risk factors include HPV infection (especially HPV-16), Epstein-Barr virus infection (mainly nasopharyngeal cancer), chewing of paan, drinking yerba mate, eating salty foods, poor oral hygiene, exposure to radiation, and industrial exposures. Treatment options include surgery, chemotherapy, targeted therapy, and/or radiation therapy.

#### CK2 in head and neck cancer

Oncomine analysis showed that *CK2α*, *CK2α’* and *CK2β* transcripts were significantly overexpressed in head and neck cancers (Table 6). In addition, Bian *et al*. using the TGCA database find genetic and transcript expression alterations in *CK2α* (21%), *CK2α’* (11%) *and CK2β* (8%) in head and neck squamous cell carcinoma cases (HNSCC). Most are increases in *CK2* transcript expression. However, in a small percentage of tumor samples, *CK2* transcript levels decreased [73]. Intriguingly, the majority of these tumor samples have alterations (up or downregulation) in the levels of transcripts of only one of the *CK2* genes. There was no data for *CK2αP* in any of the cancer types in Table 6. There were no studies in Oncomine for other types of head and neck cancer.

**Table 6:** Analysis of changes in *CK2* gene expression in head and neck cancer. P-values, fold change, rank and datasets are shown.

Gene	p-value	Fold Change	Rank (Top %)	Dataset	#Samples	Reference
**Floor of the Mouth Carcinoma**						
*CK2α*	1.69 10^−4^	2.313	7%	Pyeon Multi-cancer	84	[63]
*CK2α’*	0.011	1.99	21%	Pyeon Multi-cancer	84	[63]
*CK2β*	1.44 10^−4^	1.923	6%	Pyeon Multi-cancer	84	[63]
**Oral Cavity Squamous Cell Carcinoma**						
*CKα*	2.35 10^−6^	1.301	11%	Peng Head-Neck	57	[76]
**Nasopharyngeal carcinoma**						
*CK2α*	5.01 10^−5^	1.381	6%	Sengupta Head-Neck	41	[77]
**Oropharyngeal Carcinoma**						
*CK2α*	6.85 10^−4^	1.737	7%	Pyeon Multi-cancer	84	[63]
**Tongue Carcinoma**						
*CK2α*	1.31 10^−6^	1.735	2%	Pyeon Multi-cancer	84	[63]
*CK2α’*	1.74 10^−6^	1.836	3%	Pyeon Multi-cancer	84	[63]
*CK2β*	5.75 10^−4^	1.689	14%	Pyeon Multi-cancer	84	[63]
	3.76 10^−4^	1.409	19%	Talbot Lung	93	[78]
	0.003	1.605	23%	Estilo Head-Neck	58	[63]
**Tonsillar Carcinoma**						
*CK2β*	0.006	1.514	7%	Pyeon Multi-cancer	84	[63]

HNSCC sections show strong nuclear immunostaining of all three CK2 proteins [33, 74], correlating with elevated levels of CK2 activity in nuclear and chromatin fractions compared to cytosol fractions [31–33]. HNSCC cell lines also show increased expression levels of all three CK2 proteins [74].

**Fig 4:**
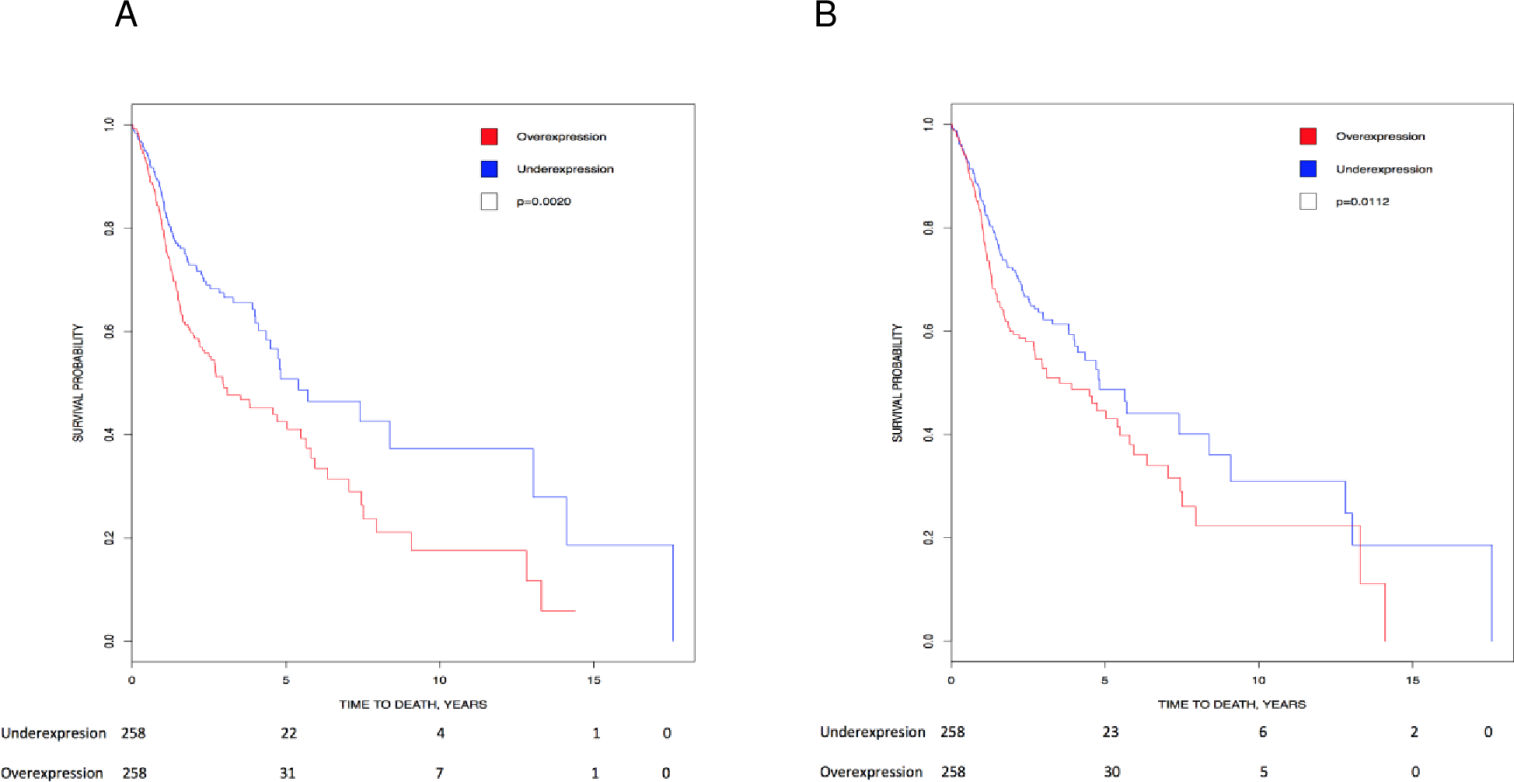
Correlation of *CK2* gene expression to overall patient survival in head and neck cancer. Patients were stratified into above-median (red line) and below-median (blue line) expression of *CK2*. (A) High levels of *CK2α’* transcript correlated with lower survival (HR = 1.53, 95% CI [1.166, 2.009]; p = 0.002). (B) High levels of *CK2β* transcript correlated with lower survival (HR = 1.411, 95% CI [1.080, 1.843]; p = 0.0112).

Kaplan-Meier analysis showed that higher expression of *CK2α’* (p = 0.002) and *CK2β* (p = 0.0112) transcripts directly correlated with lower survival in head and neck cancer patients (Fig 4). In line with these data, there is high CK2 activity (nuclear, cytosolic and chromatin bound) in head and neck cancer [31–33], which correlates with disease status and decreased patient survival [31]. Based on the data above, levels of CK2 transcripts and protein activity could be a prognostic marker for HNSCC. Therefore, CK2 could be a target for cancer therapy. Indeed, pre-clinical evidence suggests that CK2 inhibitors could be effective in HNSCC (reviewed in [16]). In addition, a pre-clinical model of feline oral squamous cell carcinoma, thought to be a good model for human head and neck cancer, show promising results when CK2α and CK2α’ are targeted with RNAi [75]. CK2 targeting is associated with low toxicity (little adverse events, with weight loss and anorexia being the most common).

### Kidney cancer

Types of kidney cancer include renal cell carcinoma (80%) (subdivided into clear cell, papillary, and chromophobe), renal pelvis carcinoma, transitional cell carcinoma (7%), Wilms tumor (develops in children under 5 years of age), and renal sarcoma (including clear cell sarcoma of the kidney, a rare form that occurs in children typically between 1–4 years of age). Renal oncocytoma is a benign renal tumor. Incidence of kidney cancer has been increasing by an average of 1.6% per year over the past decade. Kidney cancer is twice as common in men as in women, and is also more prevalent among African Americans, American Indians, and Alaska natives. Factors contributing to kidney cancer include smoking, obesity, hypertension, and particular conditions that are inherited. There is currently no standard screening test for kidney cancer. However, individuals with increased risk due to inherited conditions can be screened for kidney cancer using computed tomography (CT) and magnetic resonance imaging (MRI). Treatments include surgery, radiation therapy, chemotherapy, biological therapy, and targeted therapy.

#### CK2 in kidney cancer

Data from Oncomine revealed a significant overexpression of all three *CK2* genes in renal cancers as detailed in (Table 7). In contrast, Roelants *et al*. find a decrease in all *CK2* transcript levels of 1.5–16 times by RT-qPCR in clear cell renal carcinoma [79]. This observation on decreased transcript levels is in conflict with our analysis and that of Rabjerg *et al*. [36, 39]. Rabjerg *et al*. show increased expression of *CK2α*, *CK2α’* and *CK2β* transcripts in renal cell carcinoma by RT-qPCR in 97 patients (Rabjerg, Guerra et al. 2017)[39]. The Roelans *et al*. and Rabjerg *et al*. studies have similar demographics except for country of origin (Roelants *et al*: Grenoble, France, 53% males, 73% over 65 years, 33.3% pT3-T4, 66.7% Fuhrman grade III-IV, 20% metastasis,15 samples *versus* Rabjerg *et al*.: Denmark, 56% males, 49% over 65 years, 40% pT3-T4, 45% Fuhrman grade III-IV, 29% metastasis, 97 samples). The only difference between studies was the age of the patients. There was no data for *CK2αP* in any of the cancer types in Table 7.

**Table 7:** Analysis of changes in *CK2* gene expression in kidney cancer. P-values, fold change, rank and datasets are shown.

Gene	p-value	Fold Change	Rank (Top %)	Dataset	#Samples	Reference
**Chromophobe Renal Cell Carcinoma**						
*CK2α*	1.64 10^−5^	2.212	1%	Yusenko Renal	67	[82]
*CK2α’*	0.002	1.517	13%	Jones Renal	92	[83]
	0.012	2.130	14%	Yusenko Renal	67	[82]
**Clear Cell Renal Cell Carcinoma**						
*CK2α*	1.64 10^−5^	1.410	20%	Jones Renal	92	[83]
	1.11 10^−4^	1.444	6%	Yusenko Renal	67	[82]
*CK2β*	4.79 10^−8^	1.506	12%	Jones Renal	92	[83]
**Clear Cell Sarcoma of the Kidney**						
*CK2α*	9.5 10^−5^	1.569	3%	Cutcliffe Renal	35	[84]
**Papillary Renal Cell Carcinoma**						
*CK2α*	3.15 10^−5^	1.543	2%	Yusenko Renal	67	[82]
	5.5 10^−4^	1.360	26%	Jones Renal	92	[83]
**Renal Oncocytoma**						
*CK2α*	4.76 10^−5^	1.993	2%	Yusenko Renal	67	[82]
	2.97 10^−6^	1.36	11%	Jones Renal	92	[83]
*CK2α’*	1.11 10^−7^	1.505	7%	Jones Renal	92	[83]
	0.004	2.015	11%	Yusenko Renal	67	[82]
*CK2β*	0.005	1.424	12%	Yusenko Renal	67	[82]
**Renal Pelvis Urothelial Carcinoma**						
*CK2α*	9.74 10^−4^	1.364	19%	Jones Renal	92	[83]
*CK2β*	9.79 10^−4^	1.448	19%	Jones Renal	92	[83]
**Renal Wilms Tumor**						
*CK2α*	5.36 10^−5^	2.199	1%	Yusenko Renal	67	[82]
	4.39 10^−4^	1.417	3%	Cutcliffe Renal	35	[84]
*CK2β*	0.002	1.500	5%	Cutcliffe Renal	35	[84]
	0.004	1.607	6%	Yusenko Renal	67	[82]

Despite finding decreased levels of all *CK2* transcripts, Roelants *et al*. find increased CK2 activity, and increase in CK2α, CK2α’ and CK2β protein levels in renal cell carcinoma in 15 patients [79]. Similarly, other renal clear cell carcinoma studies show increase in CK2 activity [36, 80]. Timofeeva *et al*. find higher staining of CK2β protein in primary kidney tumors while expression of CK2α protein is unchanged [81]; and propose that CK2 activity may be upregulated in these kidney tumors, as increased expression of CK2β results in increased CK2 activity [81].

CK2 protein and transcript have diagnostic value in renal cell carcinoma. Thus, high nuclear CK2α staining correlates with late metastasis, but no other clinical variables [36]. Furthermore, high *CK2α* transcript expression correlated with high Fuhrman grade and tumor stage and, intriguingly, high *CK2α’* transcript expression and low Fuhrman grade [36].

**Fig 5:**
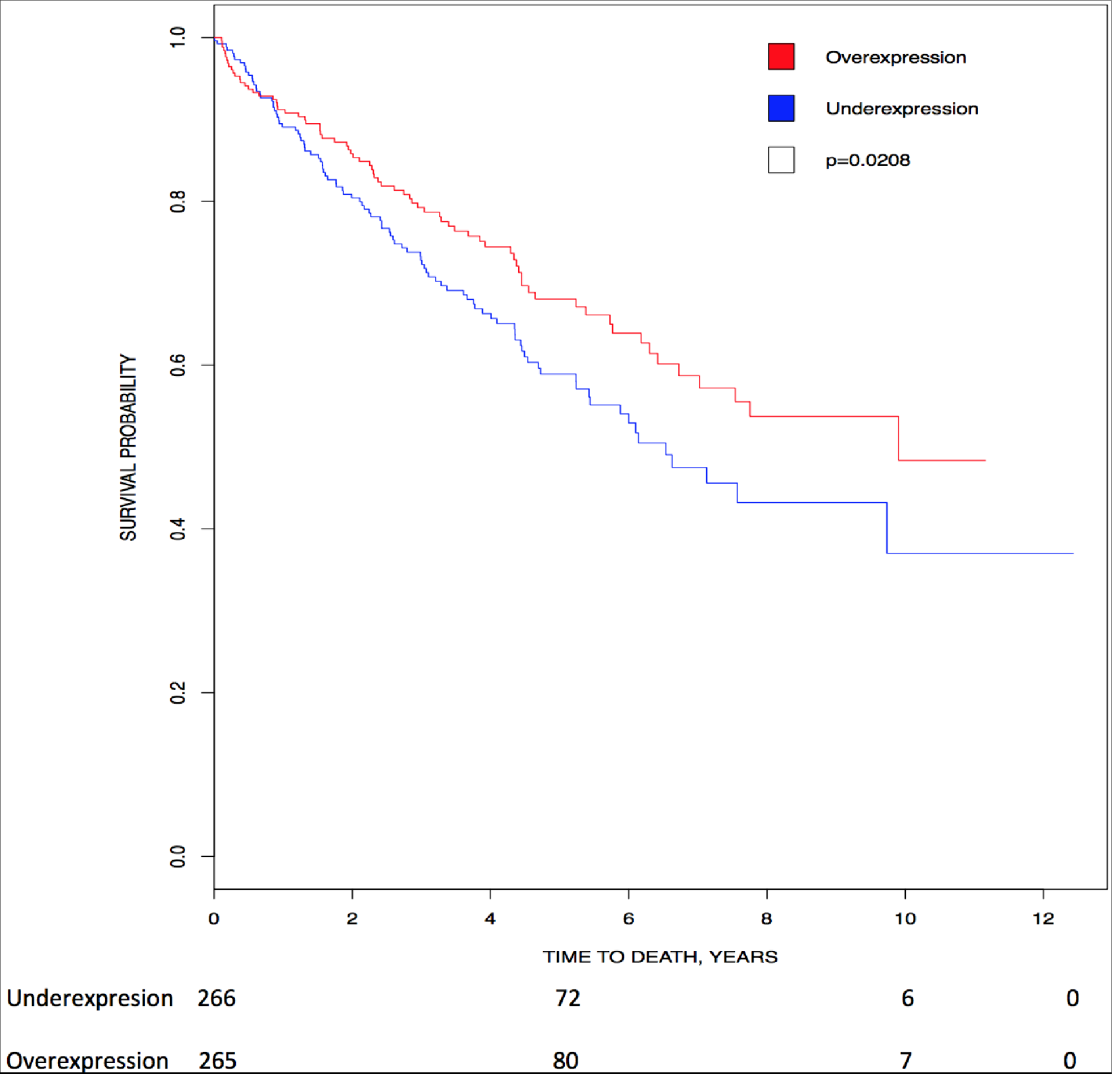
Correlation of *CK2* gene expression to overall patient survival in renal clear cell carcinoma. Patients were stratified into above-median (red line) and below-median (blue line) expression of *CK2αP*. High levels of *CK2αP* transcript correlated with higher survival (HR = 0.702, 95% CI [0.520, 0.949]; p = 0.0208).

Unexpectedly, our Kaplan-Meier analysis showed that higher expression of *CK2αP* (p = 0.0208) directly correlated with higher survival in patients with renal clear cell carcinoma (Fig 5). In addition, Rabjerg *et al*. find that high *CK2α* transcript expression and high nuclear CK2α staining correlate with poor overall survival, disease specific survival and progression free survival in patients with renal cell carcinoma [36, 39]. In summary, *CK2αP*, and CK2α transcript and protein have a prognostic value in renal cell carcinoma; and nuclear CK2α protein, *CK2α* and *CK2α*’ transcripts have a diagnosis value.

### Leukemia

Leukemia is a cancer where the bone marrow produces abnormal white blood cells known as leukemia cells, and is one of the most common childhood cancers. Such cells are non-apoptotic, and their expansion can impede proper function of normal white blood cells, red blood cells, and platelets. Leukemia can be divided into four main types, which are acute myeloid leukemia (AML), acute lymphoblastic leukemia (ALL, most common in children), chronic lymphocytic leukemia (CLL), and chronic myeloid leukemia (CML). Hairy cell leukemia (HCL) is rare (2% of all leukemia cases). Risk factors include smoking, chemical exposure such as benzene, radiation exposure, history of chemotherapy or radiation therapy, inherited or genetic conditions, blood disorders, and a family history of leukemia. There is currently no standard screening test for leukemia. Treatments include watchful waiting, chemotherapy, targeted therapy, radiation therapy, and stem cell transplant.

#### CK2 in leukemia

*CK2α’*, *CK2β* and *CK2αP* transcripts were overexpressed in ALL. In T-cell ALL (T-ALL) and B-cell ALL (B-ALL) there were conflicting results for *CK2α* (Table 8). For B-ALL, the Coustan-Smith *et al*. (Finland) and the Maia *et al*. study (USA) studies showed significant downregulation, while the Andersson *et al*. study (Sweden) showed significant upregulation of the *CK2α* transcript [85–87]. In all three studies, leukemia samples were collected from children (Coustan-Smith *et al*. from patients age 1–18y; Maia *et al*. from patients age 0.1–17y; Andersson *et al*. did not specify children’s age range). The studies did not disclose patient demographics, including sex or ethnicity. The Coustan-Smith *et al*. and the Maia *et al*. studies both used age-matched controls while the Andersson *et al*. study used adult controls. This could potentially have confounded their results as there are innate differences in immunity between the adult and pediatric population [88]. Similarly, for T-ALL, the Coustan-Smith *et al*. study and Andersson *et al*. study showed opposing findings, with the Coustan-Smith *et al*. study showing significant downregulation while the Andersson *et al*. study showed significant upregulation of *CK2α* transcript expression. As stated above, these studies focused on the pediatric population, however Andersson *et al*. did not specify the age range. There was also no mention of patient demographics. Furthermore, the Andersson *et al*. study used adult controls. Together, these could have accounted for the differences in results between the two studies. Published studies find CK2α protein levels and CK2 activity are elevated in primary B-ALL [89] and T-ALL cells [90]. CK2α’ protein levels are elevated in primary B-ALL cells [89]. CK2β protein levels elevated in primary T-ALL cells [90].

**Table 8:** Analysis of changes in *CK2* gene expression in leukemia. P-values, fold change, rank and datasets are shown. (* = childhood leukemia).

Gene	p-value	Fold Change	Rank (Top %)	Dataset	#Samples	Reference
**Pro-B Acute Lymphoblastic Leukemia**						
*CK2α’*	9.25 10^−25^	1.515	3%	Haferlach Leukemia	2096	[104]
**B-Cell Acute Lymphoblastic Leukemia (B-ALL)**						
*CK2α*	1.57 10^−4^	-1.63	5%	Maia Leukemia*	28	[85]
	0.001	-1.758	10%	Coustan-Smith Leukemia*	288	[86]
	0.004	1.528	35%	Andersson Leukemia*	127	[87]
*CK2α’*	4.54 10^−26^	1.421	5%	Haferlach Leukemia	2096	[104]
	8.31 10^−29^	1.389	6%	Haferlach Leukemia*	2096	[104]
	2.65 10^−7^	2.189	10%	Andersson Leukemia*	127	[87]
*CK2β*	6.45 10^−15^	2.130	2%	Andersson Leukemia*	127	[87]
*CK2αP*	0.004	1.528	35%	Andersson Leukemia*	127	[87]
**T-Cell Acute Lymphoblastic Leukemia (T-ALL)**						
*CK2α*	4.04 10^−4^	1.743	20%	Andersson Leukemia*	127	[87]
	0.004	-1.399	14%	Coustan-Smith Leukemia*	288	[86]
*CK2α’*	1.77 10^−7^	2.496	4%	Andersson Leukemia*	127	[87]
	1.35 10^−27^	1.576	5%	Haferlach Leukemia	2096	[104]
*CK2β*	1.44 10^−8^	2.654	2%	Andersson Leukemia*	127	[87]
*CK2αP*	0.011	1.589	33%	Andersson Leukemia*	127	[87]
**Acute Myeloid Leukemia (AML)**						
*CK2α*	0.002	1.609	27%	Andersson Leukemia*	127	[87]
*CK2α’*	1.47 10^−7^	2.030	5%	Andersson Leukemia*	127	[87]
	1.05 10^−20^	1.417	5%	Haferlach Leukemia*	2096	[104]
*CK2β*	3.26 10^−8^	2.006	4%	Andersson Leukemia*	127	[87]
	2.96 10^−4^	-1.928	5%	Stegmaier Leukemia*	87	[91]
*CK2αP*	0.002	1.609	27%	Andersson Leukemia*	127	[87]
**Chronic Lymphocytic Leukemia (CLL)**						
*CK2α*	1.34 10^−6^	-2.228	5%	Rosenwald Multi-cancer	102	[105]
	5.89 10^−34^	-1.453	7%	Haferlach Leukemia	2096	[104]
	2.06 10^−4^	-1.505	10%	Alizadeh Leukemia	120	[97]
*CK2α’*	3.13 10^−34^	1.455	8%	Haferlach Leukemia	2096	[104]
	3.82 10^−4^	-1.316	19%	Rosenwald Multi-cancer	102	[105]
*CK2β*	3.33 10^−4^	1.885	10%	Haslinger Leukemia	111	[98]
	2.81 10^−6^	-1.391	8%	Basso Lymphoma	336	[100]
	0.004	-1.381	8%	Rosenwald Lymphoma	293	[101]
**Hairy Cell Leukemia (HCL)**						
*CK2α*	2.48 10^−4^	-1.308	16%	Basso Lymphoma	336	[100]
*CK2α’*	1.42 10^−8^	-1.876	3%	Basso Lymphoma	336	[100]

In AML, Oncomine analysis showed overexpression of *CK2α*, *CK2α’* and *CK2αP*, and conflicting findings for *CK2β*. Thus, the Andersson *et al*. study (Sweden) assayed AML samples from pediatric patients, while the Stegmaier *et al*. study (USA) used adult patients [87, 91]. As above, the intrinsic differences between the pediatric and adult population may play a role in the discrepancy between the two studies for *CK2β* transcript expression levels. In this regard, there are differences in gene abnormalities between pediatric and adult patients in other cancers and diseases. For example, the frequency of prognostic markers among pediatric and adult B-ALL differ, with adult patients harboring more mutations and epigenetic modifications compared to pediatric patients [92]. Similarly, there are pediatric-adult differences in mature B-cell non-Hodgkin lymphoma and Celiac disease [93, 94]. These studies indicate that gene expression alterations may also be different among pediatric and adult AML patients, as we see here for *CK2β*. Similar to our findings, published studies also demonstrate *CK2α* transcript overexpression in AML cell lines compared to normal hematopoietic cells [95]. As for CK2 proteins, primary AML cells show both increased and decreased levels of CK2α protein and CK2 activity [95, 96]. High CK2α protein levels are a prognostic biomarker, as they correlated with low overall survival and decreased disease-free survival in AML patients [96]. However, high levels of CK2α protein do not correlate with clinical variables, including subtype [96].

CLL showed downregulation of *CK2α*, and conflicting findings for *CK2α’* and *CK2β*. For *CK2α’*, the Haferlach *et al*. study found significant upregulation, while the Rosenwald *et al*. study showed significant downregulation. The Haferlach *et al*. study was a multicenter study conducted across seven countries in 11 different study centers, while the Rosenwald *et al*. study was a single center study conducted in the United States. However, demographic information in the two studies were not provided. Haferlach *et al*. used samples from patients who had not undergone prior treatment, while 12.1% of patients in the Rosenwald *et al*. study had received prior chemotherapy treatment. Additionally, the Haferlach *et al*. used bone marrow and/or peripheral blood samples, while the Rosenwald *et al*. used only peripheral blood samples. In the control group, Haferlach *et al*. used bone marrow samples from healthy individuals or individuals without leukemia (such individuals may have a preexisting blood disorder such as hemophilia). Rosenwald *et al*. used samples in their control group from a prior study where they collected cells form tonsils, adult apheresis products, or cord blood [97]. Together, these differences across the two studies could have led to the differences in results. For *CK2β*, the Haslinger *et al* (Germany) study had 62% male patients (30–87 years; median 62.5) [98]. 62% patients had Binet stage A, 33% had stage B, and 5 patients had stage C CLL (the stage that has the worst prognosis). Additionally, 47% of patients had VH-mutations [98], which has a more favorable prognosis and less aggressive CLL disease compared to patients with unmutated VH [99]. Rosenwald *et al*. (USA) and Basso *et al*. (USA) did not disclose patient demographic or clinicopathological information [100, 101]. Thus, we do not have enough data to explain the discrepancy in the results. In contrast to our transcript level results, primary CLL cells show increased CK2α and CK2β proteins and CK2 activity [102]. These data suggest that CK2 is regulated both transcriptionally and post-transcriptionally.

In HCL, *CK2α’* was downregulated. Published data show that in Jurkat, a T-ALL cell line, *CSNK2A1P* has high transcript levels correlating with gene copy number [103].

There was no data for CML in Oncomine. Pre-clinical evidence suggests that CK2 inhibitors could be effective in treating leukemia (reviewed in [16]).

### Lymphoma

Lymphoma, a cancer of the lymphocytes, is the leading blood cancer in the USA. Lymphoma can be subdivided into Hodgkin lymphoma (defined by the presence of Reed-Sternberg cells) and non-Hodgkin lymphoma (NHL). NHLs can derive from B-cells (85%), T-cells (<15%) or NK cells (rare). NHLs can be further subdivided into high grade (fast-growing) and low grade (slow-growing).

Types of B-cell NHLs include low-grade lymphomas (follicular lymphoma, mantle cell lymphoma) and high-grade lymphomas (diffuse large B-cell lymphoma (DLBCL), Burkitt lymphoma, and primary effusion lymphoma). DLBCL can be further subdivided into activated B cell-like DLBCL, which has the lowest survival of all NHL types, and germinal center B cell-like DLBCL. The most common high grade NHL is DLBCL; the most common low grade NHL is follicular lymphoma.

T-cell NHL can include anaplastic large cell lymphoma, angioimmunoblastic T-cell lymphoma, and unspecified peripheral T-cell lymphoma, which are all high-grade lymphomas. Adult T-cell leukemia/lymphoma (ATL) is usually a highly aggressive NHL.

Risk factors for lymphoma include HIV, Epstein-Barr virus, *Helicobacter pylori*, or human T-cell leukemia/lymphoma virus type 1 (HTLV-1) infection, having a weak immune system and age. Both high and low grade NHL are treated with chemotherapy, and, increasingly, biological (stem cell transplant) or targeted therapies.

#### CK2 in non-Hodgkin lymphoma

Oncomine analysis showed mixed regulation of all three *CK2* transcripts in NHL (Table 9). There was only data for *CK2αP* in follicular lymphoma and DLBCL. The data in Oncomine for Hodgkin lymphoma was not-significant.

**Table 9:** Analysis of changes in *CK2* gene expression in non-Hodgkin lymphoma. P-values, fold change, rank and datasets are shown. (NOS = not otherwise specified).

Gene	p-value	Fold Change	Rank (Top %)	Dataset	#Samples	Reference
**Follicular Lymphoma (**low grade B-cell NHL)						
*CK2α*	3.21 10^−7^	-1.421	17%	Compagno Lymphoma	136	[108]
*CK2α’*	8.51 10^−4^	-2.263	3%	Storz Lymphoma	27	[109]
	0.004	1.341	13%	Brune Lymphoma	67	[110]
	8.94 10^−4^	-1.301	40%	Compagno Lymphoma	136	[108]
*CK2β*	4.37 10^−8^	-1.493	13%	Compagno Lymphoma	136	[108]
**Mantle Cell Lymphoma (**low grade B-cell NHL)						
*CK2α*	7.63 10^−4^	1.400	11%	Basso Lymphoma	336	[100]
*CK2β*	0.001	1.376	13%	Basso Lymphoma	336	[100]
**Diffuse Large B Cell Lymphoma NOS (DLBCL-NOS) (**high-grade B-cell NHL)						
*CK2α*	0.001	1.324	15%	Basso Lymphoma	336	[100]
	0.003	1.350	16%	Rosenwald Lymphoma	293	[101]
	0.001	-1.564	11%	Rosenwald Multi-cancer	102	[105]
**Activated B-Cell-like Diffuse Large B Cell Lymphoma (DLBCL) (**high-grade B-cell NHL)						
*CK2α*	4.29 10^−5^	1.383	18%	Compagno Lymphoma	136	[108]
*CK2β*	1.18 10^−4^	1.616	12%	Alizadeh Lymphoma	120	[97]
**Germinal Center B-Cell-like Diffuse Large B Cell Lymphoma (DLBCL) (**high-grade B-cell NHL)						
*CK2α*	0.003	-1.380	26%	Compagno Lymphoma	136	[108]
*CK2α’*	1.15 10^−5^	1.322	8%	Alizadeh Lymphoma	120	[97]
*CK2β*	0.008	1.530	23%	Alizadeh Lymphoma	120	[97]
**Burkitt’s Lymphoma (**high-grade B-cell NHL)						
*CK2α*	0.005	1.302	24%	Basso Lymphoma	336	[100]
*CK2β*	2.44 10^−4^	2.331	9%	Brune Lymphoma	67	[110]
**Primary Effusion Lymphoma (**high-grade B-cell NHL)						
*CK2α’*	0.001	-1.853	11%	Basso Lymphoma	336	[100]
**Anaplastic Large Cell Lymphoma (**high-grade T-cell NHL)						
*CK2α*	2.78 10^−6^	1.844	5%	Piccaluga Lymphoma	60	[111]
	0.01	1.437	25%	Eckerle Lymphoma	64	[112]
*CK2α’*	3.66 10^−8^	2.195	2%	Piccaluga Lymphoma	60	[111]
*CK2β*	0.003	2.002	25%	Piccaluga Lymphoma	60	[111]
**Angioimmunoblastic T-Cell Lymphoma (**high-grade T-cell NHL)						
*CK2α*	4.57 10^−4^	1.940	15%	Piccaluga Lymphoma	60	[111]
*CK2α’*	0.004	1.858	25%	Piccaluga Lymphoma	60	[111]
*CK2β*	0.006	1.549	26%	Piccaluga Lymphoma	60	[111]
**Unspecified Peripheral T-Cell Lymphoma (**high-grade T-cell NHL)						
*CK2α*	3.32 10^−5^	1.525	21%	Piccaluga Lymphoma	60	[111]
*CK2α’*	5.79 10^−4^	1.427	27%	Piccaluga Lymphoma	60	[111]
**Chronic Adult T-cell Leukemia/Lymphoma (ATL or ATLL)**						
*CK2α’*	0.011	-1.534	12%	Choi Leukemia	47	[113]
**T-cell/Histiocyte-rich B-cell Lymphoma (T/HRBCL)**						
*CK2α*	7.9 10^−4^	1.323	4%	Brune Lymphoma	67	[110]

In follicular lymphoma, the Storz *et al*. and Compagno *et al*. studies show downregulation while the Brune *et al*. study show upregulation of *CK2α’* transcript expression. Both the Storz *et al*. study and the Compagno *et al*. studies were conducted in the United States, while the Brune *et al*. study was conducted in Germany. Only the Storz *et al*. study provided demographic information, with patient ages ranging from 34 to 88. No other information was provided. All three studies used tonsil samples from healthy individuals. There is not enough information to speculate other reasons for the differences in results.

In DLBCL-NOS, the Basso *et al*. and 2002 Rosenwald *et al*. study show upregulation of *CK2α* transcript expression, while the 2001 Rosenwald *et al*. study showed downregulation. There is not enough information to speculate other reasons for the differences in results in these USA studies, as only the 2002 Rosenwald *et al*. study provided patient demographic information, with 55% of patients >60 years old (median age 63), 56% male, 37% had low risk DLBCL while 14% had high risk DLBCL, and most patients in this study (47%) had centroblastic monomorphic histologic subtype.

With respect to protein levels, CK2α and CK2β protein staining is stronger in follicular lymphoma, Burkitt’s lymphoma, and DLBCL, and in lymphoma cell lines, showing both nuclear and cytoplasmic staining [106]. However, the intensity of staining did not correlate with tumor grade, at least in follicular lymphoma [106]. In addition, CK2α and CK2β staining was mantle cell lymphoma tissue sections [107]. Interestingly, overexpression of *CK2α* in lymphocytes of transgenic mice results in T cell lymphomas, suggesting an oncogenic role for CK2α [17–19].

### Monoclonal gammopathies

Myeloma is the 3^rd^ most common blood cancer and is caused by abnormal plasma cells, called myeloma cells, that build up in the bone marrow and cause damage to the solid bone. Accumulation of myeloma cells leading to macroscopic lesions in multiple bone sites is termed “multiple myeloma”. In addition to affecting bones, myeloma can also damage other tissues and organs. Types of myeloma include smoldering myeloma (or asymptomatic myeloma), symptomatic myeloma, and plasma cell leukemia, a rare and aggressive form of myeloma with poor prognosis. Elevated levels of antibodies, known as M proteins, produced by myeloma cells in blood, urine, and organs leads to a condition called monoclonal gammopathy of undetermined significance (MGUS). While this condition can be asymptomatic, it can become associated with other diseases or progress to myeloma or other plasma cell malignancies (eg, lymphoma or amyloidosis). Risk factors for myeloma include older age, history of MGUS or having isolated plasmacytoma of the bone. Demografic (age, race), genetic and environmental factors (e.g. radiation, herbicides) play a role in the development of MGUS. There is currently no standard screening test for myeloma. Myeloma treatments include chemotherapy, targeted therapy, biological therapy, corticosteroid treatment, and stem cell transplant with high-dose chemotherapy. In certain instances, radiation or surgery to repair damaged bones may be needed.

#### CK2 in monoclonal gammopathies

Oncomine analysis showed significant overexpression of *CK2α*, *CK2α’* and *CK2β* (Table 10). MGUS showed conflicting data for *CK2α*, where the Agnelli *et al*. study showed underexpression while the Zhan *et al*. study showed overexpression [114, 115]. The Zhan *et al*. study was conducted in the USA (45% females, 36% over 65 years, 84% white race) and the Agnelli *et al*. study was conducted in Italy (~40% females, median age 67). We cannot explain the discrepancy. There was no data for *CK2αP* in any of the types listed in Table 10.

**Table 10:** Analysis of changes in *CK2* gene expression in monoclonal gammopathies. P-values, fold change, rank and datasets are shown.

Gene	p-value	Fold Change	Rank (Top %)	Dataset	#Samples	Reference
**Monoclonal Gammopathy of Undetermined Significance (MGUS)**						
*CK2α*	1.18 10^−6^	1.594	4%	Zhan Myeloma 3	78	[115]
	6.61 10^−5^	-1.619	1%	Agnelli Myeloma 3	158	[114]
*CK2α’*	0.003	1.225	26%	Zhan Myeloma 3	78	[115]
*CK2β*	0.001	1.308	21%	Zhan Myeloma 3	78	[115]
**Smoldering Myeloma**						
*CK2α*	3.36 10^−9^	1.763	1%	Zhan Myeloma 3	78	[115]
*CK2α’*	0.002	1.378	32%	Zhan Myeloma 3	78	[115]
*CK2β*	1.73 10^−4^	1.779	22%	Zhan Myeloma 3	78	[115]
**Plasma Cell Leukemia**						
*CK2α*	0.001	1.881	8%	Agnelli Myeloma 3	158	[114]
*CK2β*	6.4 10^−4^	1.461	7%	Agnelli Myeloma 3	158	[114]

Interestingly, multiple myeloma primary cells and cell lines had elevated levels of CK2α and CK2β proteins and CK2 activity [116], and CK2α and CK2β staining was darker in multiple myeloma tissue sections [107]. However, in MGUS, CK2α and CK2β staining was no different from normal hematopoietic cells [107]. Therefore, it is plausible that *CK2* transcript levels are elevated in myeloma.

### Liver cancer

Liver cancer most commonly begins in hepatocytes giving rise to hepatocellular carcinoma (also known as primary liver cancer). Other liver cancer types such as fibrolamellar carcinoma, hemangiosarcoma, and hepatoblastoma are rare. Hepatocellular carcinoma is most commonly caused by cirrhosis of the liver due to alcohol abuse, hepatitis B, hepatitis C, hemochromatosis, steatohepatitis, obesity, and diabetes. There is currently no standard screening test for liver cancer. Treatments include surgery, liver transplant, ablation, embolization, radiation therapy, chemotherapy, and targeted therapy.

#### CK2 in liver cancer

Oncomine analysis showed significant overexpression of *CK2α* and *CK2β* in hepatocellular carcinoma (Table 11). In agreement with our analysis, two other groups find *CK2α* transcripts are upregulated in hepatocellular carcinoma [40, 117]. *CK2α* transcript levels correlate with increasing tumor grade [40], and with elevated levels of CK2α protein in hepatocellular carcinoma samples [40, 117]. Therefore, increases in CK2α protein may be due to *CK2α* transcript upregulation. As for subcellular localization, CK2α staining is found in membrane and cytoplasm [117].

**Table 11:** Analysis of changes in *CK2* gene expression in liver cancer. P-values, fold change, rank and datasets are shown.

Gene	p-value	Fold Change	Rank (Top %)	Dataset	#Samples	Reference
**Hepatocellular Carcinoma**						
*CK2α*	9.93 10^−45^	1.801	6%	Roessler Liver 2	445	[119]
	1.94 10^−5^	1.774	9%	Roessler Liver	43	[119]
*CK2β*	1.69 10^−50^	1.630	4%	Roessler Liver 2	445	[119]
	1.53 10^−6^	1.720	6%	Roessler Liver	43	[119]
	1.07 10^−8^	1.417	8%	Chen Liver	197	[120]
	0.006	1.371	20%	Wurmbach Liver	75	[119, 121]

Importantly, high CK2α protein staining correlates with histological grade, distant metastasis and tumor stage [117].

**Fig 6:**
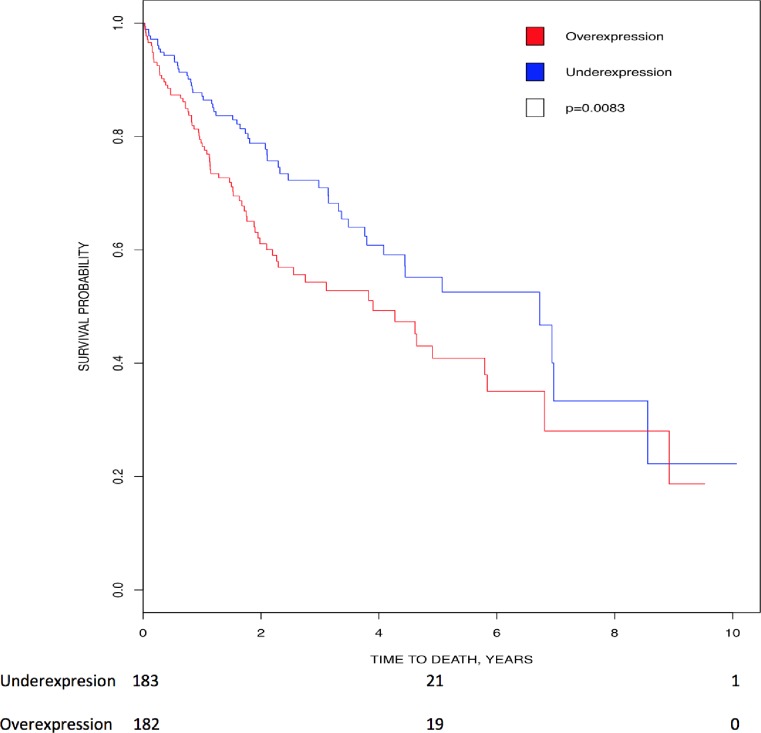
Correlation of *CK2* gene expression to overall patient survival in liver cancer. Patients were stratified into above-median (red line) and below-median (blue line) expression of *CK2*α’. High levels of *CK2α’* transcript correlated with lower survival (HR = 1.592, 95% CI [1.123, 2.256]; p = 0.0083).

Kaplan-Meier analysis showed that higher expression of *CK2α’* (p = 0.0083) directly correlated with lower survival in liver cancer (Fig 6). In addition, patients with elevated *CK2α* transcript [40] and protein staining [117] had a lower survival rate. These authors find that *CK2α* transcript overexpression is an independent prognostic factor (survival) [40, 117].

All together these data show that, CK2α and CK2α’ transcripts and CK2α protein have prognostic value, and CK2α transcript and protein have diagnostic value for hepatocellular carcinoma.

In addition to potential treatment of hepatic tumors with CK2 inhibitors, there is a potential role for CK2 inhibition in preventing steatohepatitis and subsequently associated hepatocellular carcinoma, as the CK2 phosphorylation site in Sirtuin 1 is important in the pathophysiology of obesity and hepatic steatosis [118].

### Mesothelioma

Mesothelioma is a rare type of cancer that forms from cells of the mesothelium. Mesothelioma is subdivided based on location of origin into pleural (75%), peritoneal (10–20%), pericardial (1%) and testicular (< 1%). Mesothelioma is also classified according to their compromised cell type into epithelial (50–70%), sarcomatoid (10–20%), and biphasic (mixed epithelial and sarcomatoid; 20–35%). Patients with epithelial mesothelioma have the highest survival rate due to better response to treatment. Risk factors include exposure to asbestos, even for a short amount of time. Other risk factors include radiation exposure, intrapleural thorium dioxide, inhalation of other fibrous silicates, or inheriting a germline mutation on the BAP1 gene. Following diagnosis, only about 40% of patients survive past the first year. There is no standard screening test for mesothelioma, especially for those who were exposed to asbestos. Treatment options include surgery, radiation therapy, chemotherapy, immunotherapy, and/or intraoperative intraperitoneal chemotherapy.

#### CK2 in mesothelioma

Oncomine analysis showed significant overexpression of *CK2α* and *CK2β* transcripts in malignant pleural mesothelioma (Table 12). These findings were consistent with a recently published study, where they also find an overexpression of *CK2α* transcript, and elevated staining of CK2α protein in malignant pleural mesothelioma [122]. There was no data for *CK2αP* in mesothelioma.

**Table 12:** Analysis of changes in *CK2* gene expression in mesothelioma. P-values, fold change, rank and datasets are shown.

Gene	p-value	Fold Change	Rank (Top %)	Dataset	#Samples	Reference
**Malignant Pleural Mesothelioma**						
*CK2α*	2.76 10^−6^	2.194	2%	Gordon Mesothelioma	54	[123]
*CK2β*	7.96 10^−7^	1.485	2%	Gordon Mesothelioma	54	[123]

**Fig 7:**
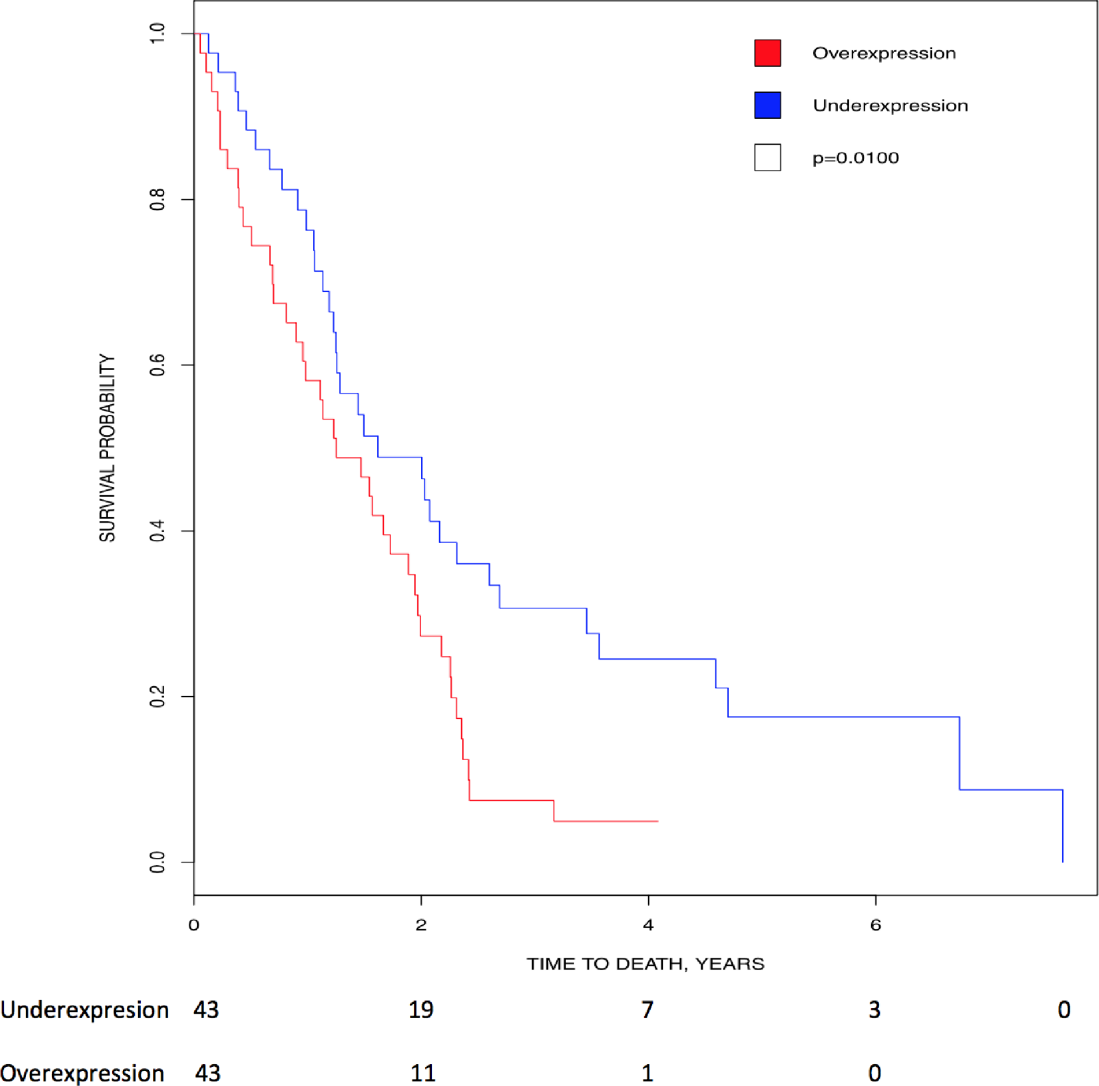
Correlation of *CK2* gene expression to overall patient survival in mesothelioma. Patients were stratified into above-median (red line) and below-median (blue line) expression of *CK2αP*. High levels of *CK2αP* transcript correlated with lower survival (HR = 1.873, 95% CI [1.153, 3.041]; p = 0.01).

Kaplan-Meier analysis showed that higher expression of *CK2αP* (p = 0.01) directly correlated with lower survival in mesothelioma, suggesting its prognostic value in this cancer (Fig 7).

### Parathyroid cancer

Parathyroid cancer affects any of the four parathyroid glands. Both benign and malignant parathyroid tumors can lead to hyperparathyroidism, where excess parathyroid hormone (PTH) is produced leading to over-absorption of dietary calcium in the intestines, and movement of stored calcium in the bones into blood. These events ultimately lead to hypercalcemia, where amount of calcium in the blood rises above normal levels. Risk factors for parathyroid adenoma include rare inherited disorders such as familial isolated hyperparathyroidism and multiple endocrine neoplasia type 1 syndrome. Treatment options include minimally invasive surgery to remove the tumor itself or treating the hypercalcemia (gallium nitrate, calcitonin, IV fluids, or drugs that prevent breakdown and reabsorption of bones). Chemotherapy and radiation therapy are not very effective in preventing parathyroid cancer recurrence. Furthermore, radiation therapy can instead increase risks of developing parathyroid adenoma.

#### CK2 in parathyroid overgrowth

Oncomine analysis showed significant overexpression of *CK2α* transcript in three types of parathyroid overgrowth (hyperplasia and benign), as well as an overexpression of *CK2α*’ transcript in parathyroid hyperplasia (Table 13). There was no data for *CK2αP* in any of the types in Table 13.

**Table 13:** Analysis of changes in *CK2* gene expression in parathyroid overgrowth. P-values, fold change, rank and datasets are shown.

Gene	p-value	Fold Change	Rank (Top %)	Dataset	#Samples	Reference
**Parathyroid Hyperplasia**						
*CK2α*	0.005	1.629	9%	Morrison Parathyroid	61	[124]
*CK2α’*	0.008	1.506	13%	Morrison Parathyroid	61	[124]
**Non-Familial Multiple Gland Neoplasia**						
*CK2α*	0.003	1.678	9%	Morrison Parathyroid	61	[124]
**Parathyroid Gland Adenoma**						
*CK2α*	0.003	1.750	12%	Morrison Parathyroid	61	[124]

### Thyroid cancer

Thyroid cancer occurs in the thyroid gland, where thyroid follicular cells normally produce thyroid hormone which regulates bodily functions such as body temperature, weight, blood pressure, and heart rate. The thyroid gland also consists of C cells which secrete calcitonin, which is important in calcium homeostasis in the body. There are four types of thyroid cancer, including papillary, follicular, medullary, and anaplastic thyroid cancer. Papillary thyroid cancer is the most common among the four, while anaplastic thyroid cancer remains the most aggressive with the lowest cure rates. Risks for thyroid cancer include genetic conditions (eg, familial medullary thyroid cancer, multiple endocrine neoplasia type 2A syndrome, multiple endocrine neoplasia type 2B syndrome), and history of irradiation to the head and neck in childhood. There is no current standard screening test, other than neck palpation during annual primary care visits. Diagnosis is usually achieved using ultrasound of the neck and fine needle biopsy. Definitive treatment for thyroid cancer is achieved by thyroidectomy (surgical removal of the thyroid).

#### CK2 in thyroid cancer

Oncomine analysis showed significant underexpression of *CK2α* in papillary carcinoma (Table 14). There was no data for *CK2αP*.

**Table 14:** Analysis of changes in *CK2* gene expression in thyroid cancer. P-values, fold change, rank and datasets are shown.

Gene	p-value	Fold Change	Rank (Top %)	Dataset	#Samples	Reference
**Thyroid Gland Papillary Carcinoma**						
*CK2α*	0.008	-1.361	15%	He Thyroid	18	[126]

Guo *et al*. found higher CK2α transcript levels and protein staining (in particular nuclear staining) in all four thyroid carcinoma subtypes (papillary, follicular, medullary, and anaplastic) [125]. Furthermore, higher nuclear CK2α protein staining was associated with lymph node metastasis and tumor stage and EMT markers, suggesting that CK2α staining can have diagnostic value [125]. This indicated a potential role for targeting CK2α for thyroid cancer treatment.

### Sarcoma

Sarcoma develops from abnormal cells of mesenchymal origin. This can include muscle, bone, fat, and/or vascular tissues. There are more than 50 types of sarcoma, but the most common types include liposarcoma (adipose tissue), synovial sarcoma (synovial lining cells in joints), and leiomyosarcoma (smooth muscle). Rhabdomyosarcoma is the most common in children. Risk factors for sarcoma include inherited conditions such as retinoblastoma, Li-Faumeni syndrome, familial adenomatous polyposis, neurofibromatosis, Werner syndrome and tuberous sclerosis, and also chemical and/or radiation exposure. There is no current standard screening test for sarcomas, and they are usually diagnosed using imaging techniques such as CT and MRI scans. Additionally, removal of a tissue sample is needed to confirm the diagnosis. Treatment options include surgery, radiation therapy, and/or chemotherapy.

#### CK2 in sarcoma

Overall, Oncomine analysis revealed significant overexpression of *CK2α*, and mostly underexpression of *CK2α’* in sarcoma. There was overexpression of *CK2β* in pleiomorphic liposarcoma and underexression in dedifferentiated liposarcoma. There were conflicting results for *CK2α’* in leiomyosarcoma (Table 15). However, leiomyosarcoma samples in the Quade *et al*. study was obtained specifically from patients with leiomyosarcoma of the uterus. Detwiller *et al*. obtained samples from patients with leiomyosarcoma in any body region, although they did not specify. Control samples in the Quade *et al*. study were uterus samples from healthy individuals, while control samples in the Detwiller *et al*. study were soft tissue samples (not specified) from healthy individuals. Thus, these differences in sample source and type may have contributed to the different findings. There was no data for *CK2αP* in all types listed in Table 15.

**Table 15:** Analysis of changes in *CK2* gene expression in sarcoma. P-values, fold change, rank and datasets are shown.

Gene	p-value	Fold Change	Rank (Top %)	Dataset	#Samples	Reference
**Dedifferentiated Liposarcoma**						
*CK2α’*	2.49 10^−4^	-1.599	11%	Barretina Sarcoma	158	[128]
*CK2β*	0.007	-1.815	5%	Detwiller Sarcoma	54	[129]
**Fibrosarcoma**						
*CK2α*	7.85 10^−4^	1.975	7%	Detwiller Sarcoma	54	[129]
*CK2α’*	0.009	-1.464	11%	Detwiller Sarcoma	54	[129]
**Malignant fibrous histiocytoma (Pleomorphic Undifferentiated Sarcoma)**						
*CK2α’*	0.011	-1.534	14%	Detwiller Sarcoma	54	[129]
**Leiomyosarcoma**						
*CK2α’*	0.002	1.392	1%	Quade Uterus	24	[130]
	1.49 10^−4^	-2.153	2%	Detwiller Sarcoma	54	[129]
**Myxofibrosarcoma**						
*CK2α’*	0.001	-1.448	19%	Barretina Sarcoma	158	[128]
**Pleomorphic Liposarcoma**						
*CK2α*	0.003	1.422	6%	Detwiller Sarcoma	54	[129]
*CK2α’*	0.003	-1.693	4%	Detwiller Sarcoma	54	[129]
	0.001	-1.494	12%	Barretina Sarcoma	158	[128]
*CK2β*	5.13 10^−5^	1.334	11%	Barretina Sarcoma	158	[128]
**Synovial Sarcoma**						
*CK2α*	1.28 10^−4^	1.801	5%	Detwiller Sarcoma	54	[129]

**Fig 8:**
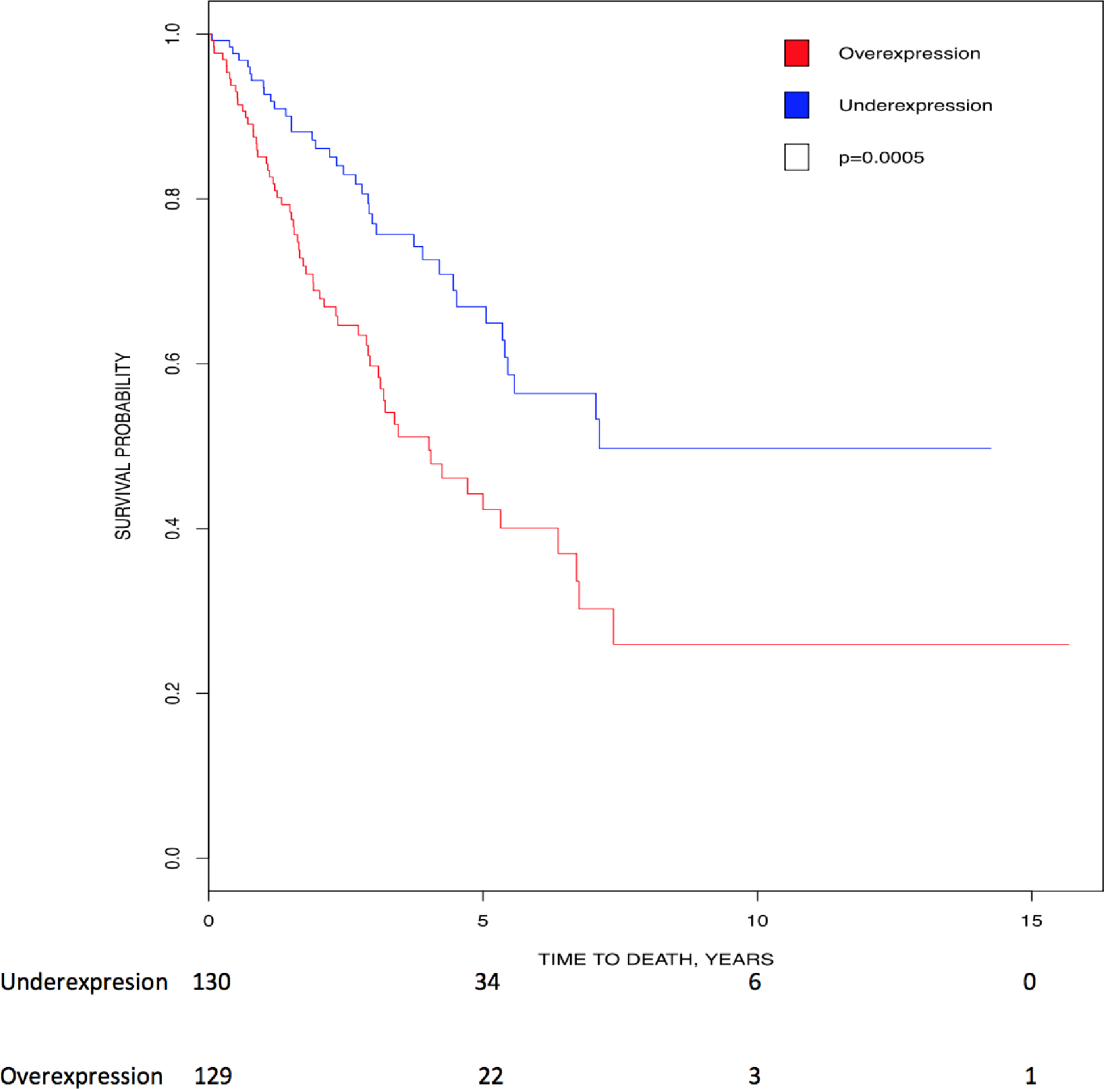
Correlation of *CK2* gene expression to overall patient survival in sarcoma. Patients were stratified into above-median (red line) and below-median (blue line) expression of *CK2α*. High levels of *CK2α* transcript correlated with lower survival (HR = 2.038, 95% CI [1.353, 3.071]; p = 0.0005).

Kaplan-Meier analysis showed that higher expression of *CK2α* transcript (p = 0.0005) directly correlated with lower survival in sarcoma, suggesting its prognostic value in this cancer type (Fig 8).

Takahashi *et al*. found overexpression of CK2α and CK2β protein in human osteosarcoma cell lines when compared to normal cells [127]. Furthermore, knockdown of either CK2α or CK2β with siRNA or incubation with CX-4945 led to prevention of osteosarcoma cell proliferation. However, CX-4945 did not inhibit cell proliferation of human mesenchymal stem cells, indicating a role for CK2 in osteosarcoma cell proliferation. *In vivo* results in xenograft mouse models found that CX-4945 inhibited osteosarcoma growth [127]. Together, these indicate a role for CK2 inhibition in osteosarcoma treatment.

### Skin cancer

Skin cancer includes melanoma and non-melanoma skin cancer (basal cell carcinoma and squamous cell carcinoma). Melanoma is the most fatal form of skin cancer and arises from melanocytes. On the other hand, non-melanoma skin cancer usually responds well to treatment and is rarely metastatic. A risk factor for skin cancer is the presence of benign melanocytic skin nevus, more commonly known as moles and freckles. Other risk factors include fair skin, high exposure to natural (sun) or artificial UV light, history of blistering sunburns, and history (family or personal) of melanoma or atypical moles. The current standard screen for melanoma is a visual examination of the skin. Treatments include surgery, chemotherapy, targeted therapy, biological therapy, and radiation therapy.

#### CK2 in melanoma

Oncomine analysis revealed significant overexpression of all three *CK2* genes as detailed in the Table below (Table 16). There was no data for *CK2αP* in any of the types listed in Table 16.

**Table 16:** Analysis of changes in *CK2* gene expression in skin cancer. P-values, fold change, rank and datasets are shown.

Gene	p-value	Fold Change	Rank (Top %)	Dataset	#Samples	Reference
**Benign Melanocytic Skin Nevus (moles and freckles)**						
*CK2α*	0.002	5.016	10%	Talantov Melanoma	70	[133]
*CK2α’*	0.004	1.566	13%	Talantov Melanoma	70	[133]
*CK2β*	8.28 10^−4^	1.797	8%	Talantov Melanoma	70	[133]
	1.02 10^−5^	1.754	2%	Haqq Melanoma	37	[134]
**Skin Basal Cell Carcinoma**						
*CK2α*	5.15 10^−4^	1.453	6%	Riker Melanoma	87	[135]
**Skin Squamous Cell Carcinoma**						
*CK2α*	1.02 10^−7^	2.081	1%	Riker Melanoma	87	[135]
**Melanoma**						
*CK2α*	9.11 10^−4^	7.026	17%	Talantov Melanoma	70	[133]
*CK2α’*	1.91 10^−4^	-2.533	3%	Riker Melanoma	87	[135]
*CK2β*	9.30 10^−5^	2.478	10%	Talantov Melanoma	70	[133]
	0.004	2.198	14%	Haqq Melanoma	37	[134]

Interestingly, ninety percent of melanomas with *CK2α* transcript upregulation had mutations in the most prevalent genes mutated in melanoma: *BRAF*, *NRAS* and/or *NF1* [131]. Moreover, these authors found increased CK2α protein expression in melanoma cell lines when compared with normal human melanocytes [131]. Intriguingly, CK2α staining and transcript levels are elevated in melanomas with NRAS Q61 mutations but not in melanomas with NRAS G12 mutations [132]. These suggest a potential for the use of CK2 inhibitors to treat this cancer. We reviewed the use of CK2 inhibitors in combination with current therapies for melanoma in [16].

### Testicular cancer

90% of testicular cancer derive from germ cells, and the remaining 10% derive from supporting cells (Leydig cells, Sertoli cells). Germ cell-related testicular cancer can be subdivided equally into seminomas and non-seminomas. Non-seminoma testicular cancer is subdivided into teratomas, embryonal carcinomas (3–4%), mixed germ cell tumors, and yolk sac tumors. Typically, non-seminoma tumors grow faster, present with an earlier diagnosis age, and have a lower relative 5-year survival rate. In contrast, seminomas have a higher survival rate largely due to the fact that they are highly sensitive to radiation therapy and chemotherapy. Germ cell tumors can also occur outside of the gonads due to defects in embryonic development. There are many risk factors for testicular cancer, including cryptorchidism, Turner syndrome, androgen insensitivity syndrome, Klinefelter’s syndrome, low fertility, and family history. Treatment options for testicular cancer include surgery, radiation therapy, and chemotherapy.

#### CK2 in testicular cancer

Oncomine analysis revealed significant downregulation of all three *CK2* genes and *CK2αP* in both seminomatous and non-seminomatous testicular cancer (Table 17). *CK2αP* was also downregulated in testicular seminoma. There was no data for *CK2αP* in any of the other types listed in Table 17.

**Table 17:** Analysis of changes in *CK2* gene expression in testicular cancer. P-values, fold change, rank and datasets are shown. Not Otherwise Specified was a category in Oncomine however, in the case of Seminoma and teratoma they are the same as the category above.

Gene	p-value	Fold Change	Rank (Top %)	Dataset	#Samples	Reference
**Testicular Seminoma**						
*CK2α*	0.003	-1.645	22%	Sperger Others	74	[142]
*CK2α’*	0.009	-1.640	27%	Sperger Others	74	[142]
*CK2β*	0.012	2.607	9%	Skotheim Testis	30	[143]
*CK2αP*	0.003	-1.645	22%	Sperger Others	74	[142]
**Seminoma, Not Otherwise Specified**						
*CK2α*	1.21 10^−5^	-2.224	13%	Korkola Seminoma	107	[144]
*CK2α’*	8.56 10^−11^	-8.257	1%	Korkola Seminoma	107	[144]
*CK2β*	2.11 10^−6^	-2.180	10%	Korkola Seminoma	107	[144]
**Testicular Teratoma**						
*CK2α’*	0.006	-4.093	7%	Skotheim Testis	30	[143]
*CK2β*	0.009	-2.471	9%	Skotheim Testis	30	[143]
**Teratoma, Not Otherwise Specified**						
*CK2α*	3.56 10^−9^	-3.805	4%	Korkola Seminoma	107	[144]
*CK2α’*	5.12 10^−12^	-9.860	1%	Korkola Seminoma	107	[144]
*CK2β*	3.93 10^−8^	-2.791	6%	Korkola Seminoma	107	[144]
**Embryonal Carcinoma, Not Otherwise Specified**						
*CK2α*	5.98 10^−8^	-2.666	6%	Korkola Seminoma	107	[144]
*CK2α’*	6.42 10^−12^	-9.596	1%	Korkola Seminoma	107	[144]
*CK2β*	1.59 10^−6^	-2.349	11%	Korkola Seminoma	107	[144]
**Mixed Germ Cell Tumor, Not Otherwise Specified**						
*CK2α*	1.25 10^−7^	-2.302	10%	Korkola Seminoma	107	[144]
*CK2α’*	4.33 10^−12^	-7.593	3%	Korkola Seminoma	107	[144]
*CK2β*	6.47 10^−7^	-2.377	12%	Korkola Seminoma	107	[144]
**Yolk Sac Tumor, Not Otherwise Specified**						
*CK2α*	0.014	-1.524	30%	Korkola Seminoma	107	[144]
*CK2α’*	1.21 10^−9^	-6.599	1%	Korkola Seminoma	107	[144]
*CK2β*	9.08 10^−5^	-2.123	15%	Korkola Seminoma	107	[144]

To our knowledge, there is no data on the CK2 protein levels in testicular cancer, or on the mechanism by which CK2 dysregulation could be affecting testicular cancer. However, we have knowledge on CK2 levels in normal testicular tissue and in reproductive organ development. Specifically, *CK2α*’ and *CK2β* transcript, protein levels and CK2 activity are highest in mouse testicles compared to other mouse tissues [5, 136, 137]. In mice and *Xenopus laevis*, *CK2* transcript levels are high in germ cells (reviewed in [137]). Importantly, *CK2α’* is required for male gametogenesis, as knockout of *CK2α’* mice are infertile and have oligospermia and globozoospermia (i.e. spermatozoa having round heads) [5]. In addition, the natural CK2 inhibitor Emodin causes hypospermatogenesis and apoptosis of mouse germ cells [138]. These phenotypes could be due in part to germ cell chromatin breakdown, a risk factor for testicular cancer [139]. Potentially, CK2 could be acting on two chromatin remodeling proteins, CK2 Target protein 2 (CKT2) and testis-specific protein, Y-encoded (TSPY) [140, 141]. Since *CK2α’* is required for sperm differentiation in mice, downregulation of *CK2α’* in testicular cancer could delay and/or prevent germ cell differentiation, and possibly divert sperm development towards tumor formation.

## Conclusion

Our goal was to determine the extent to which *CK2* genes could be significantly up- or down-regulated in cancers we did not study in our previous publication [9], and to determine whether the *CK2* gene expression levels in these cancers correlated with overall patient survival. Here we show that there is dysregulation of *CK2* transcripts in a number of these cancer types that was not reported previously, and that over- and under- expression of *CK2* transcripts was cancer-type specific. In addition, the prognostic value of *CK2* genes was also cancer-type specific as both over- and under-expression correlated with poor survival. In addition, we have compared our data to published results on CK2 gene and protein expression.

In Table 18, we summarized the analysis from Oncomine and of the literature. In many cases these results are robust, as several studies showed the same up- or down-regulation for the *CK2* transcript (e.g. up to 4 studies for *CK2α* and *CK2β* increase in hepatocellular carcinoma) (Table 18). The fact that independent studies showed similar data gives strength to the conclusions reached. As we have previously found in other cancers [9], we found cancers with significant upregulation of all three *CK2* genes, such as bladder cancers, and tumors with mixed up- and down- regulation of *CK2* transcripts. In solid tumors with mixed up- and down-regulation, *CK2α’* was almost always downregulated (e.g. sarcomas and melanoma), as it was in breast, ovarian and pancreatic cancers [9]. In addition, we found a third pattern of dysregulation of *CK2* genes: significant downregulation of all three *CK2* transcripts. Finding all three *CK2* genes downregulated (e.g. Barrett’s esophagus and testicular cancer) was unexpected, particularly since CK2α/α’ downregulation leads to decreased proliferation (reviewed in [16, 22, 23]). This is a novel finding in CK2α/α’ biology, and requires further study to determine whether CK2α/α’ protein levels are equally decreased and, if so, which hallmark of cancer [145] could be affected by CK2α’ protein decrease. In contrast to the little data on CK2α’ downregulation, CK2β downregulation is already linked to cancer progression. In particular, to increased epithelial to mesenchymal transition [146]. Further investigation as to whether *CK2* gene downregulation contributes to cancer development in these cancer types, including animal model studies, will address the relevance of this finding. One possibility in testicular cancer is that aberrant expression of *CK2α’* and *CK2β* could cause a dysfunction in gametogenesis leading to subfertility; as subfertility has been linked to increased risk of developing testicular cancer [139].

**Table 18:** Expression of CK2 transcript and proteins in cancers. This table summarizes some of our Oncomine data on upregulation (red arrow, ↑) or downregulation (dark blue arrow, ↓) of CK2 transcripts. Multiple arrows (e.g.: ↑↑) indicate the number of studies showing similar conclusions. We also included published data on upregulation (orange arrow, ↑) or downregulation (light blue arrow, ↓) of CK2 transcripts and protein levels. Each arrow indicates an independent study. (n.s.) Not significant; (-) No data; (ND) Not determined (*) Diagnostic potential; Abbreviations as defined in the text.

Cancers	*CK2 Transcripts*				CK2 Proteins			
	*α*	*α’*	*β*	*αP*	α	α’	β	CK2 activity
**Bladder**								
Superficial Bladder Carcinoma	↑↑	↑↑	↑↑	n.s.				
Invasive Bladder Carcinoma	↑↑	↑	↑↑	n.s.	↑*[45] ↑* [46]			
Bladder Carcinoma (NOS)	↑[44]	ND	ND	ND	↑*[44]			
**CNS cancers**								
Astrocytoma	↑↑ [51] ↑[50]/↓↓ [50]	n.s. ↑ [51]	↑↑ [51]	-	↑[50]↓ [50]			↑ [51]
Glioblastoma (Astrocytoma Grade IV)	↑↑↑↑[49] ↑[38] ↑ [51] ↑[50]/↓ ↓[38] ↓[49] ↓[50]	↑ [51]/↓↓	↑↑ [51]/↓	n.s.	**↑***[50]↑[38]↑[55]↑ [Dubois, 2016 #532]↑[54]]/ ↓[38]			↑ [51]
Anaplastic Oligodendroglioma	n.s.↑ [51]	↑↑ [51]	↑↑ [51]	n.s.				↑ [51]
Anaplastic Oligoastrocytoma	↑	↓	↑	n.s.				
**Cervical**								
Cervical	↑	↑	↑	-				
**Esophageal**								
Barrett’s Esophagus	↓	↓	↓↓	↓				
Esophageal Adenocarcinoma	↓↓	↑/↓	↓	↓				
Esophageal Carcinoma	ND	ND	↑* [66]/↓[66]	ND			**↑***[66]/↓[66]	
**Gastric**								
Gastric Intestinal Type Adenocarcinoma	↑↑	↑/↓	↑	↑	↑*[29]		↑*[30]	
Diffuse Gastric Adenocarcinoma	↑	↓	n.s.	n.s.	↑*[29]		↑*[30]	
Gastric Mixed Adenocarcinoma	↑↑	n.s.	n.s.	n.s.	↑*[29]			
**Head and Neck**								
Floor of the Mouth Carcinoma	↑	↑	↑	-				
Oral Cavity Squamous Cell Carcinoma	↑	n.s.	n.s.	-				
Nasopharyngeal Carcinoma	↑	n.s.	n.s.	-				
Oropharyngeal Carcinoma	↑	n.s.	n.s.	-				
Tongue Carcinoma	↑	↑	↑↑↑	-				
Tonsillar Carcinoma	n.s.	n.s.	↑	-				
H&N squamous cell carcinoma	↑/↓ [73]	↑*/↓[73]	↑*/↓[73]	-	↑ [74] ↑ [33]	↑ [74] ↑ [33]	[74]	↑* [31] ↑ [32] ↑ [33]
**Kidney**								
Chromophobe Renal Cell Carcinoma	↑	↑↑	n.s.	-				
Clear Cell Renal Cell Carcinoma	↑↑↑*[39] ↑*[36]/↓[79]	n.s. **↑**[39] ↑*[36]/ ↓[79]	↑↑[36]/↓[79]	-	↑[79]↑* [36] n.s. [81]	↑[79];	↑[79] ↑ [81]	↑ [79] ↑ [80]↑ [36]
Clear Cell Sarcoma of the Kidney	↑	n.s.	n.s.	-				
Papillary Renal Cell Carcinoma	↑↑	n.s.	n.s.	-				
Renal Oncocytoma	↑↑	↑↑	↑	-				
Renal Pelvis Urothelial Carcinoma	↑	n.s.	↑	-				
Renal Wilms Tumor	↑↑	n.s.	↑↑	-				
**Leukemia**								
Pro-B ALL	n.s.	↑	n.s.	-				
ALL (B-Cell)	↑/↓↓	↑↑↑	↑	↑	↑[89]	↑[89]		↑↑[89] ↑ [150]
ALL (T-Cell)	↑/↓	↑↑	↑	↑	↑[90]		↑[90]	↑[90]
AML	↑↑	↑↑	↑/↓	↑	↑[95, 96]↓[95, 96]			↑[95, 96]↓[95, 96]
CLL	↓↓↓	↑/↓	↑/↓↓	-	↑[102]		↑[102]	↑[102]
HCL	↓	↓	n.s.	-				
**NHL**								
FL (B-cell, Low Grade)	↓	↑↓↓	↓	n.s.	↑[106]		↑[106]	
Mantle (B-cell, Low Grade)	↑	n.s.	↑	-	↑[107]		↑[107]	
DLBCL-NOS (B-cell, High Grade)	↑↑↓	n.s.	n.s.	n.s.	↑[106]		↑[106]	
DLBCL-ABC (B-cell, High Grade)	↑	n.s.	↑	-				
DLBCL-GCB (B-cell, High Grade)	↓	↑	↑	-	↑[106]		↑[106]	
Burkitt’s Lymphoma (B-cell, High Grade)	↑	n.s.	↑	-	↑[106]		↑[106]	
PEL (B-cell, High Grade)	n.s.	↓	n.s.	-				
ALCL (T-cell, High Grade)	↑↑	↑	↑	-				
AITL (T-cell, High Grade)	↑	↑	↑	-				
PTCL-U (T-cell NHL High Grade)	↑	↑	n.s.	-				
ATL or ATLL	n.s.	↓	n.s.	-				
T/HRBCL	↑	n.s.	n.s.	-				
MGUS	↑/↓	↑	↑	-	n.s. [107]		n.s. [107]	
Smoldering Myeloma	↑	↑	↑	-				
Plasma Cell Leukemia	↑	n.s.	↑	-				
Multiple Myeloma	n.s.	n.s.	n.s.	-	↑[107] ↑ [116]	n.s. [116]	↑[107] ↑ [116]	↑[116]
**Liver**								
Hepatocellular Carcinoma	**↑↑↑***[40] ↑[117]	n.s.	↑↑↑↑	n.s.	↑↑↑[40] ↑*[117]			
**Mesothelioma**								
Malignant Pleural Mesothelioma	↑↑[122]	n.s.	↑	-	↑[122]			
**Parathyroid**								
Parathyroid hyperplasia	↑	↑	n.s.	-				
Non-Familial Multiple Gland Neoplasia	↑	n.s.	n.s.	-				
Parathyroid Gland Adenoma	↑	n.s.	n.s.	-				
**Thyroid**								
Thyroid Carcinoma	↑↑[151]	n.s.	n.s.	-	↑[151]			
**Sarcoma**								
Dedifferentiated Liposarcoma	n.s.	↓	↓	-				
Fibrosarcoma	↑	↓	n.s.	-				
Malignant fibrous histiocytoma	n.s.	↓	n.s.	-				
Leiomyosarcoma	n.s.	↑/↓	n.s.	-				
Myxofibrosarcoma	n.s.	↓	n.s.	-				
Pleomorphic Liposarcoma	↑	↓↓	↑	-				
Synovial Sarcoma	↑	n.s.	n.s.	-				
**Skin cancers**								
Benign Melanocytic Skin Nevus	↑	↑	↑↑	-				
Basal Cell Carcinoma	↑	n.s.	n.s.	-				
Squamous Cell Carcinoma	↑	n.s.	n.s.	-				
Melanoma	↑↑[131] ↑ [132]	↓	↑↑	-	↑[131] ↑ [132]			.
**Testicular**								
Testicular Seminoma	↓	↓	↑	↓				
Seminoma NOS	↓	↓	↓	-				
Testicular Teratoma	n.s.	↓	↓	-				
Teratoma NOS	↓	↓	↓	-				
Embryonal Carcinoma NOS	↓	↓	↓	-				
Mixed Germ Cell Tumor NOS	↓	↓	↓	-				
Yolk Sac Tumor NOS	↓	↓	↓	-				

The Oncomine analysis led to additional insights. *CK2αP* was upregulated or downregulated following the prevalent pattern of up- or down-regulation of the other *CK2* transcripts. Similarly, *CK2αP* follows the same pattern in lung, breast and colorectal cancers [9]. The importance of *CK2αP* deregulation in cancer is to be determined. We found altered expression of *CK2* transcripts already in benign lesions (e.g. parathyroid overgrowth and benign Melanocytic Skin Nevus), and also in Barrett’s esophagus (that increases the risk of developing esophageal cancer). This is similar to what is previously found in benign colon adenomas [9]. This suggests a role in cancer initiation in these cancer types. In NHL, low grade (slow-growing) lymphomas (e.g. FL) had downregulation of CK2 transcripts while in high grade NHL (more aggressive) there was upregulation of CK2 transcripts. This suggest differential transactivation of the *CK2* genes or differential stability of the transcripts may be playing a role in high grade NHL.

In Table 18 we also summarized the literature on CK2 protein levels in diverse cancers. In general, the increases in *CK2* transcripts paralleled increases in CK2 protein expression levels, nuclear localization, and/or activity; with the exception of B-cell ALL, AML, CLL and Follicular lymphoma (Table 18). Therefore, we cannot directly infer the levels of CK2 proteins in human cancer from studies of transcript levels, particularly in blood cancers. These data support the notion that there are multiple layers of regulation of CK2 expression that need to be addressed in future studies.

A number of studies show that higher CK2 protein levels, nuclear localization, and/or activity have diagnostic value, that is, correlate with clinicopathological factors (mostly with higher tumor grade and tumor stage; and sometimes with metastasis and invasion) (Table 18, asterisks). Only one exception, were increase *CK2α’* transcript correlated with low grade clear cell renal cell carcinoma Rabjerg, Guerra et al. 2017). The significance of this finding needs to be addressed. Interestingly, the diagnostic value of CK2 proteins is linked mostly to higher overall staining, nuclear staining or CK2 activity. However, in the case of astrocytomas, it is cytoplasmic CK2 staining that has a diagnostic value [50]. Importantly, in some cancers, increases in both *CK2* transcript and protein expression have diagnostic value (e.g. Clear Cell Renal Cell Carcinoma [36]) (Table 18, asterisks). This suggests that both CK2 protein and transcript levels could be used as diagnostic biomarkers. Future in depth analyses will determine the extent to which CK2 transcript and protein levels correlate and have diagnostic value in these cancers. Ultimately, changes in the overall and relative levels of CK2 proteins and of the composition of the CK2 heterotetramer will affect diverse cellular functions (e.g. proliferation, epithelial to mesenchymal transition). These effects could be mediated through changes in the activation of CK2-dependent signaling pathways or expression of downstream genes as reviewed in [16].

In Table 19, we summarized the value of CK2 as a prognostic marker. In most cancers, overexpression of one or two *CK2* gene transcripts led to lower survival (e.g. gastric cancer, sarcoma, head and neck and liver cancer) (Table 19). In addition, overexpression of the pseudogene *CK2αP* led to lower survival in cervical cancer and mesothelioma. However, similar to lung adenocarcinoma [9], overexpression of *CK2αP* in renal clear cell carcinoma led to increased survival. The significance of this finding is unclear, as we know little about the function of *CK2αP*. Importantly, there was a tight parallel between the prognostic value of CK2 transcripts and proteins, furthering the notion that abnormal levels of CK2 can be a useful prognostic marker (Table 18). Intriguingly, the levels of *CK2α’* were not statistically significant in the Oncomine analysis while they were a prognostic marker in liver cancer. This lack of significance in Oncomine analysis may be due to a high standard deviation in *CK2α’* transcript levels in liver cancer samples. This result indicates that the prognostic value of a *CK2* transcript (and perhaps of CK2 protein) needs to be investigated independently of whether the average expression level is significantly different or not from control samples.

**Table 19:** Prognostic value of CK2 transcript and protein in cancer. High *CK2* transcript correlate with higher (↑) or lower (↓) patient survival. We also included published data on correlation of high CK2 transcripts and protein (indicated as (p)) levels with lower survival (light blue arrow, ↓). Each arrow indicates an independent study. (#) = increased CK2 activity correlates with decreased patient survival [31].

High levels of Expression of:	*CK2α*	*CK2α’*	*CK2β*	*CK2αP*
**Glioblastoma**	↑ / ↓ [38]	n.s	n.s	n.s
**Cervical Cancer**	n.s	n.s	n.s	↓(RNA)
**Gastric Cancer**	↓↓(p) [29]	n.s	↓↓(p) [30]	n.s
**Head and Neck Cancer (#)**	n.s	↓	↓	n.s
**Renal Clear Cell Carcinoma**	↓↓(p) [36, 39]	n.s	n.s	↑
**AML**	↓(p) [96]	-	-	-
**Liver Cancer**	↓ [40] ↓(p) [117]	↓	n.s	n.s
**Mesothelioma**	n.s	n.s	n.s	↓
**Sarcoma**	↓	n.s	n.s	n.s

In this study, as we did for [9], we have followed an unbiased approach to analyze and report all the data available to date in the databases. This has resulted in unique studies which show that both *CK2* over- and under- expression have significance in cancer. There are intrinsic limitations to the database analyses. For example, not all cancer types are included in the databases searched, and, in some cases, the number of patients studied is not large enough to find significant differences in transcript levels or in survival rates. There are also inherent factors (algorithms used in microarray analysis, reference genes used for normalization, the age of the samples, preservation, *etc*) which could limit the outcome of the analyses or contribute to the discrepancies between studies. Those discrepancies should be resolved when additional independent studies are performed. A broader limitation to our study is that Oncomine does not include information on *CK2* gene mutations which might also affect overall patient survival.

There is a need for cancer biomarkers (diagnostic, prognostic and predictive), and for therapeutic targets and treatments to address cancer incidence and mortality in the world (Table 20). Our study advances the notion that *CK2* transcript expression is a potential diagnostic and prognostic biomarker for specific types of cancer. However, a number of validation steps are needed before *CK2* gene expression has an impact in clinical practice [147–149]. This includes whether CK2 transcripts and proteins could be used alone or in combination as diagnostic and prognostic factors. In addition, future studies could begin to address the predictive value of CK2 to select those patients who are more likely to benefit from a particular cancer treatment.

**Table 20:** Cancer statistics overview. 2016 USA statistics of the individual cancer types are analyzed and rank (USA and worldwide), incidence, mortality, 5-year survival rate (%), and estimated expenditure in billions (2014 data) are included. N/A indicates no data was available.

	Rank (USA)	Rank (worldwide)	Incidence	Mortality	5-year survival rate (%)	Estimated cost (billion)
**Bladder**	5	9	76,960 (4.6%)	16,390 (2.8%)	77.5	4.1
**CNS**	16	17	23,770 (1.4%)	16,050 (2.7%)	33.8	4.9
**Cervical**	21	7	12,990 (0.8%)	4,120 (0.7%)	67.5	1.3
**Esophageal**	18	8	16,910 (1.0%)	15,690 (2.6%)	18.4	1.6
**Gastric**	15	5	26,370 (1.6%)	10,730 (1.8%)	30.4	1.8
**Head and neck**	N/A	15	48,330 (2.9%)	9,570 (1.6%)	64.0	N/A
**Kidney**	9	12	62,700 (3.7%)	14,240 (2.4%)	73.7	4.8
**Leukemia**	11	11	60,140 (3.6%)	24,400 (4.1%)	59.7	5.9
**Non-Hodgkin Lymphoma**	7	10	72,580 (4.3%)	20,150 (3.4%)	70.7	13.4
**Myeloma**	14	23	30,330 (1.8%)	12,650 (2.1%)	48.5	N/A
**Liver**	13	6	39,230 (2.3%)	27,170 (4.6%)	17.5	N/A
**Mesothelioma**	N/A	N/A	3,000 (0.02%)	N/A	N/A	N/A
**Parathyroid**	N/A	N/A	<100	N/A	89	N/A
**Thyroid**	N/A	N/A	56,870 (3.4%)	2,010 (0.3%)	98.2	N/A
**Sarcoma**	N/A	27	15,000 (1.0%)	6,200 (1.0%)	50	N/A
**Skin (Melanoma)**	6	19	76,380 (4.5%)	10,130 (1.7%)	91.5	2.9
**Testicular**	25	26	8,720 (0.5%)	380 (0.1%)	95.4	N/A
